# Prediction of nucleic acid binding probability in proteins: a neighboring residue network based score

**DOI:** 10.1093/nar/gkv446

**Published:** 2015-05-04

**Authors:** Zhichao Miao, Eric Westhof

**Affiliations:** Architecture et Réactivité de l'ARN, Université de Strasbourg, Institut de biologie moléculaire et cellulaire du CNRS, 15 Rue Descartes, 67000 Strasbourg, France

## Abstract

We describe a general binding score for predicting the nucleic acid binding probability in proteins. The score is directly derived from physicochemical and evolutionary features and integrates a residue neighboring network approach. Our process achieves stable and high accuracies on both DNA- and RNA-binding proteins and illustrates how the main driving forces for nucleic acid binding are common. Because of the effective integration of the synergetic effects of the network of neighboring residues and the fact that the prediction yields a hierarchical scoring on the protein surface, energy funnels for nucleic acid binding appear on protein surfaces, pointing to the dynamic process occurring in the binding of nucleic acids to proteins.

## INTRODUCTION

Protein–nucleic acid (NA) interactions play crucial roles in a wide variety of functions ranging from transcription, translation, post-transcriptional/-translational modification and post-transcriptional/-translational regulation. An important step in understanding the recognition mechanism is to locate the functional residues on the RNA-/DNA-binding proteins (RBP/DBP) in an unbiased and systematic manner. This need is becoming even more critical with the massive outcome of biological sequence data (1) and the growing numbers of non-canonical protein–RNA interactions, such as chromatin regulatory factors that were not initially thought to be DBP (2). Although computational prediction of functional residues is an established field, the question is far from being settled. The difficulty is compounded by the amazing diversity in protein recognition folds as well as in RNA conformational states. Our main and first aim here is to derive a binding score for the probability of NA binding to a protein on the sole basis of the physicochemical and evolutionary features that can be directly derived from the protein structure. Afterward the score is used in order to apply the score for the prediction of NA-binding residues to proteins with unknown binding properties and to cases where the protein structure is unknown.

Previously, a significant number of prediction studies (3–6) focusing on NA-binding residues have been carried out. However, the relationship between binding sites and physicochemical or evolutionary features has not been clarified. On one hand, some previous approaches (5) have combined physicochemical features and evolutionary features, but used ‘black box’ approaches with loose and hidden relationships between features. On the other hand, programs based on physicochemical (7,8) and evolutionary (9) features are not competitive in terms of prediction accuracy (10). Furthermore, the approach of NA binding has not been unified in prediction. RNA- and DNA-binding residue predictions are always treated as different problems or trained with different datasets within the same framework (5,11,12). Finally, the current predictions formulate the problem as a binary classification problem (binding or not binding) that tends to overemphasize comparisons between residues in different proteins (Supplementary Note 1).

Here, on the basis of the coordinates of protein structures, we show that the protein electrostatics potential, accessible surface area (ASA) and sequence conservation entropy (CE) can be used in predicting RNA-binding residues achieving both high and stable accuracies on different datasets. Because protein binding residues recognize synergistically NA residues through a network of interactions, our approach combines linearly those central features using a neighboring network scoring. The network scoring attempts to monitor the real neighboring network relationships between residues and is continuous on the protein surface. The binding sites happen therefore as contact patches. The final prediction scores not only infer the likelihood for RNA binding but also show the presence of energy funnels on the protein surfaces pointing to the underlying dynamic process during protein–NA binding complex formation. Interestingly, this approach, named RBscore, also achieves high accuracies on DBP without further training, which indicates that the common and basic driving forces for RNA/DNA binding of proteins were adequately captured. A web server of RBscore (http://ahsoka.u-strasbg.fr/rbscore/) based on the new prediction approach is available. The web server allows a user to derive the RBscore for a protein with a known structure or from a single sequence of a protein.

## MATERIALS AND METHODS

### Datasets

RBP structures were obtained from NPIDB database (13) (Jan 2014) with resolution better than 3.5 Å and R factor <0.3 as criteria. PISCES (14) and TMalign (15) were used to check sequence and structural similarity. Sequence identity <25% and TMscore <0.7 were used as thresholds to remove redundancy. In the results, 130 protein chains (named as R130) were collected as a training set, while other 117 protein chains (R117) were taken as an independent test set. The 130 proteins in the R130 training set are annotated by protein names, and several typical RNA-binding domains are found and annotated. RNA-binding domains have also been checked for redundancy. Some previous works, especially sequence-based predictors that only consider sequence identity to remove redundancy of the datasets, cannot guarantee the absence of homology between the training and test datasets. In such cases, the resulting datasets would lead to a situation of training and test with similar data included, and the models do not have predictive ability. Besides, a test set of 381 DBP (D381) was also prepared in the same way but with sequence identity <25%, resolution better than 3.0 Å and R factor <0.3. Since D381 is only used for test, structural similarity was not considered.

A further 14 RBP and 11 DBP datasets were collected from previous programs to assess prediction accuracy, including BindN_R107 (107RBP) and BindN_D62 (62DBP) from BindN+ (5), PPRInt_R86 (86 RBP) from PPRInt (16), RNABindR_R144 (17), RNABindR_R147 (18), RNABindR_R44 and RNABindR_R111(6) from RNABindR/RNABindRPlus, meta2_R44 from (4), aaRNA_R67, aaRNA_R141 and aaRNA_R205 from aaRNA (19), Sungwook_R267 and Sungwook_R727 from (20), Shandar_D140 from (21), Susan_D56 from (22), DBindR_D374 from DBindR (23), DISPLAR_D428 from DISPLAR (24), DNABINDPROT_D54 from DNABINDPROT (25), PreDNA_D224 from PreDNA (26), metaDBSite_D232 and metaDBSite_D316 from metaDBSite (27), SDCPred_D159 from SDCPred (28).

Some unreasonable cases were excluded from the assessment datasets: (i) the presence of a DBP in an RBP set (PDB ID 1a1v); (ii) superseded PDB structures; (iii) peptides shorter than 20 residues; (iv) weak and uncertain NA binding proteins including those with less than three binding residues; (cascade complex as an example in Supplementary Note 2) (v) PDB chains containing only Cα atoms; (vi) proteins constituted by two separate short peptides.

### Binding residue definition

In previous studies (5,16–18,29,30), NA-binding residues are always defined as residues that have at least one NA atom in protein contact within a distance cutoff. The different distance cutoffs that used to define NA binding sites in previous programs led to ambiguity in assessment. Here, 3.5 Å was used as a distance cutoff to define binding sites in the training set. In total, 3.5 to 6 Å with 0.5 Å as step were used as hierarchical thresholds to define binding residue in test sets (see Supplementary Note 2). Besides, an NA-binding residue always requires ASA change (ΔASA > 0 Å^2^) upon complexation with NA. ASA is measured by NACCESS (31) with default parameters.

### Assessment of accuracy

All previous prediction methods treat the binding site prediction as a classification problem. And all the residues in all the proteins are compared together as binding or non-binding. Receiver Operating Characteristic (ROC) curve together with Area Under Curve (AUC) is always used as criterion for accuracy, since it is a classical assessment for machine-learning classifier (32). Nevertheless, for the prediction of NA-binding residues on a given protein, it is not necessary to compare with all other residues on all other proteins, since different proteins have different affinities for NA. NA-binding residues only need to be more favorable to NA than non-binding residues of the same protein. Therefore, since the accuracy of prediction on a given protein can be assessed by AUC, the accuracy of a set of proteins should average accuracies of all proteins (see Supplementary Note 1). We suggest the weighted arithmetic mean of AUC (wAUC) and mean of AUC (mAUC) as two criteria of accuracy for a set of proteins:

\begin{equation*} {\rm wAUC} = \frac{{\sum {{\rm AUC}(i) \times {\rm len}(i)} }}{{\sum {{\rm len}(i)} }} \end{equation*}

\begin{equation*} {\rm mAUC} = \frac{{\sum {{\rm AUC}(i)} }}{N} \end{equation*}

For a protein *i*, AUC(*i*) is its AUC value and len(*i*) is length of the protein, while *N* is the number of proteins in a dataset. We call the AUC that compare all the residues in a dataset together as total AUC (tAUC) and still use it as a reference for comparison. However, even a high tAUC does not necessary imply a high accuracy on each of the proteins when wAUC and mAUC are low, since tAUC overestimates unnecessary comparisons between residues of different proteins.

### Representation of the physicochemical features and of the overall score

Three features, solvation energy, electrostatics potential (Q) and sequence CE, were measured to predict binding residues. The program DMS (33) was used with default parameters to define surface grids on protein surface before calculating the features. NACCESS (31) was applied with default parameters to calculate absolute ASA, the solvation energy is represented by a weighted ASA of the residue. The electrostatic potentials were measured by APBS (8) together with pdb2pqr (34,35), both with default parameters. To better represent the distribution of electrostatics, electrostatic potential of the surface grids was calculated and counted into 10 bins from −20 to 20 (KbT/ec), resulting in 10 counts. Electrostatics score is a linear combination of these 10 counts. For sequence CE, HHblits (36) was used (with –e 1e-10) to search and align homologous sequences, while Weblogo (37) was used to calculate Shannon entropy (38) and width of alignment. (For each position in a multiple sequence alignment, number sequences that have a non-gapped residue in an aligned position are divided by total sequence number of the multiple sequence alignment.) Thus, in total, there are 13 (1+10+2) feature values per residue. A feature score is assigned to a residue by a linear combination of all features:

\begin{equation*} \begin{array}{*{20}l} {E_{{\rm feat}} = w_{aa} \times {\rm ASA} + } \\ {\sum\limits_{10} {w_i \times {\rm count}_{{\rm ELEC}} (i) + w_{{\rm CE}} \times {\rm CE} + w_{{\rm width}} \times {\rm width} + C_{aa} } } \\ \end{array} \end{equation*}

ASA is the accessible surface area of the residue and *w*_*aa*_ is a residue-type-dependent weighing factor (this is required because ASA is strongly affected by the residue side chain and is thus related to residue type). *w_i_* are the weighing factors for the 10 counts of electrostatics distribution and countELEC(*i*) is the count of charged grids in the *i*th bin. Score for electrostatics is a linear combination of the 10 bins weighted by *w_i_*. CE and width are CE and alignment width values (a high CE implies that the residue is less likely to change and a high alignment width implies that the residue is less likely to be a gap). Both *w*_CE_ and *w*_width_ are positive weighing factors. *C_aa_* is a residue-type-dependent constant that reflects the binding tendency to RNA. In total, there are 52 weighing factors for the feature score (provided in Supplementary Table S1) and the feature score for a residue requires 14 of them.

### Neighboring network and scoring approach

#### Spatial neighborhood

NA-binding residues generally happen as a patch on the protein surface (7,39). Therefore, the score of an NA-binding residue should reflect the proximity of other NA-binding residues. Surface grids are defined by DMS (33). Two residues are defined as surface neighbors when a grid pair of the two residues is within 1 Å. Two non-neighbor residues with a neighbor residue in common are considered as indirect neighbors. Since indirect neighbors that are too far away may have little influence on the target residue, a limit of 18 Å Cα distance from the target residue is set for effective indirect neighbors.

#### Sequence neighborhood

Some residues are neighbors not because protein folding brings them together to form the binding interface but because they are linked by peptide bonds or local hydrogen bonds in the folded protein structure. Spatial and sequence neighborhoods have very different effects on the NA-binding feature of a given residue. Therefore, neighbor residues (*j*) are classified into three groups according to their sequence distance to the target residue (*i*):

\begin{equation*} |i - j|\left\{ {\begin{array}{*{20}l} {1,\;{\rm linked\;by\;peptide\;bond}} \\ {2 - 4,\;{\rm linked\;by\;local\;hydrogen\;bonds\;to\;form\;helix\;structure}} \\ { >4,\;{\rm residues\;gather\;up\;by\,other\;reason(forming\;interface})} \\ \end{array}} \right. \end{equation*}

Furthermore, neighbor residues with high scores are more likely to influence the target residue when the target residue also has a high score. Thus, the effects of high scored neighbors should be accentuated while low scored neighbors should be alleviated. Accordingly, neighbors can also be classified as high score neighbors when they have feature scores higher than the target residue and vice versa for low score neighbors. In this way, there are 6 (3×2) neighbor types.

For the final score of prediction, we describe a neighboring network based scoring approach to linearly integrate the features here. Because direct neighbor residues have a stronger influence on the target residue than indirect neighbors, direct neighbors are defined as the first layer of network while indirect neighbors as the second layer (see Supplementary Figure S1). The prediction score, Epred or more simply the ‘RBscore’, is defined as a combination of feature score of the target residue and the averaged neighboring feature scores of the two neighboring layers:

\begin{equation*} \begin{array}{*{20}l} {E_{{\rm pred}} = E_{{\rm feat}} + \frac{{\sum\nolimits_{{\rm direct\;neighbors}} {u_{aa} \times f_{{\rm neighbors\;type}} \times E_{{\rm feat}} } }}{{N_{{\rm direct\;neighbors}} }}} \\ { + \frac{{\sum\nolimits_{{\rm indirect\;neighbors}} {v_{aa} \times g_{{\rm neighbors\;type}} \times E_{{\rm feat}} } }}{{N_{{\rm indirect\;neighbors}} }}} \\ \end{array} \end{equation*}

*u_aa_* and *v_aa_* are residue-type-dependent weighing factors that imply different residue neighbors may have different effects on the target residue. *N* is the number of neighbors used to average the effects of the environment. *f* and *g* are weighing factors according to different neighbor types. There are 52 (20 for *w_aa_* and *u_aa_* each, 12 for neighbor types *f* and *g*) weighing factors for neighboring network. Hence, the final score includes 104 parameters (52 for feature score, 52 for neighboring network) in total and is a linear combination of all these features.

### Training of the parameters

The parameters are trained on the R130 dataset with 5-fold cross-validation and optimized with simulated annealing based Monte Carlo sampling to maximize the wAUC value. The best model is taken as the prediction model.

### Support vector machine based approach

As a reference, a support vector machine (SVM) based approach similar to other machine-learning methods was adopted (5,16,40). The position specific scoring matrix (PSSM) is generated by PSI-BLAST (41) program against Swiss-Prot (42) sequence database. An 11-mers (five sequential neighbors on each side of target residue) slide window is used to represent local information of a residue. The input information that is directly adopted from PSSM includes 220 (11×20) integer values. The SVMlight (43) was used to construct SVM classifier. RNA binding sites are defined as residue within 3.5 Å of any RNA atom. A 5-fold cross-validation was used to train on the 130 RBP training set.

### Energy funnel measure

The X-axis in the energy funnel plot is the minimum distance from RNA/DNA to a protein residue. For each residue, we measure the distance between all atoms and all NA atoms, with the minimum of the distances considered as the distance between the residue and the NA. Supplementary Figure S2 shows a simple scheme of hierarchical distances between RNA and residues in a protein.

### Homology modeling test

Alignments used to build homologous models were generated by TMalign (15) between the bound and unbound structures. Structure models used in prediction were built by MODELLER (44) based on the unbound state structures as templates and the alignments. Datasets for the homology modeling test are adopted from (45), DR_bind1 (46), DRNA (47), OPRA (48), Protein–RNA docking benchmark 1.0 (49), Protein–RNA docking benchmark I (50) and II (51), DBD-Hunter (52), Protein–DNA docking benchmark (53), DISPLAR (24) and DNABINDPROT (25).

## RESULTS

### Accuracy comparison with SVM

Current prediction methods for NA-binding residues normally compare all residues in all proteins together to measure the area under the ROC curve (AUC) for assessment. However, the comparison between residues of different proteins is not necessary. To avoid a biased assessment, we first measure the AUC of each protein and assess the prediction accuracy of a dataset with the weighted mean of AUC (wAUC) or mean of AUC (mAUC) (see Supplementary Note 1 and Materials and Methods for descriptions). An SVM-based approach similar to previous studies (5,6,16) was built alongside RBscore, as a reference, sharing the same training (R130) and test (R117) datasets (see Materials and Methods). A 5-fold cross-validation was carried out on the training set and the best model was used for tests. The results of the cross-validation can be found in Supplementary Table S2. Both the approaches were tested on 14 RBP datasets and 11 DBP datasets.

**Figure 1.**
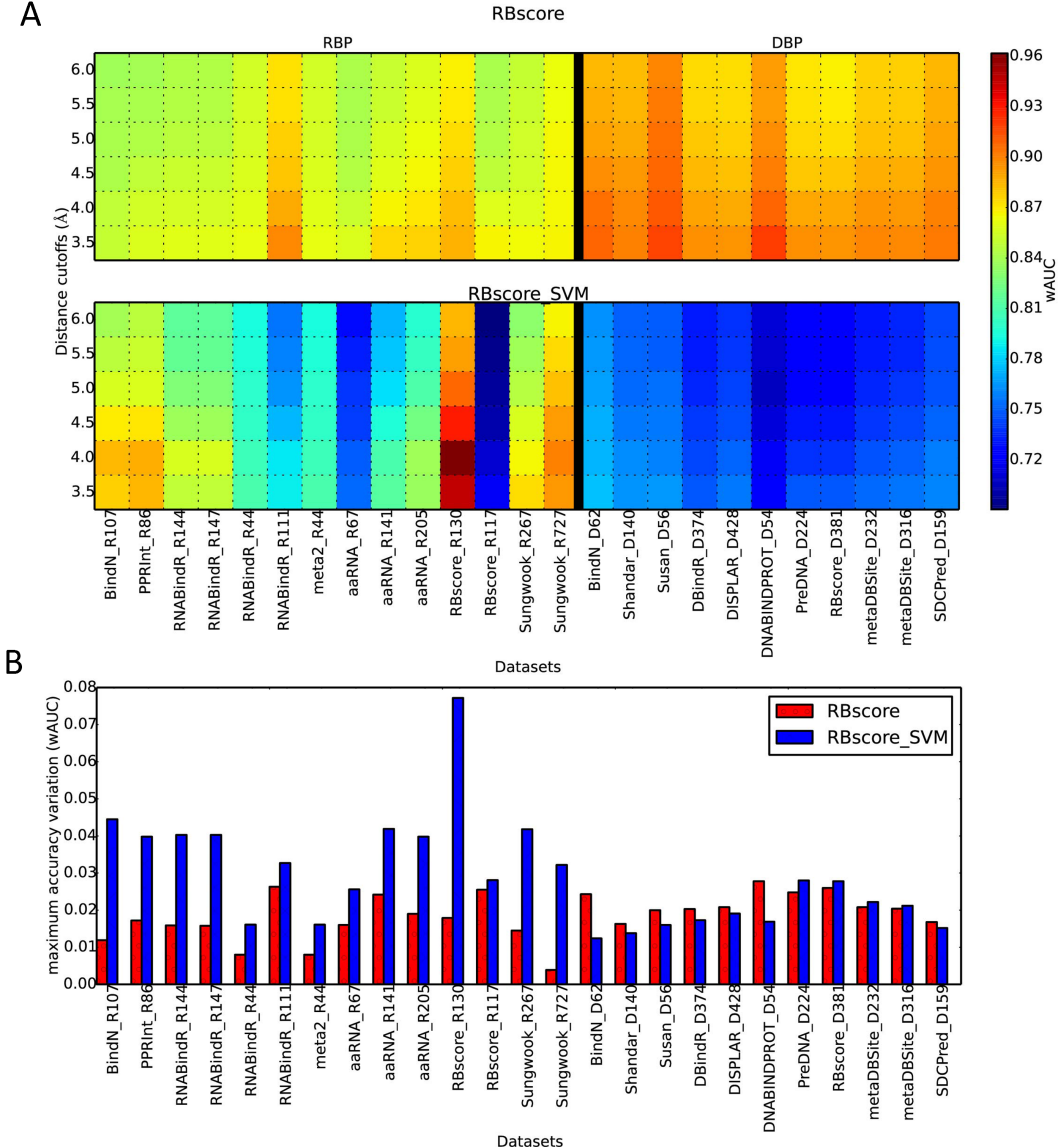
Prediction accuracy comparison between RBscore and the SVM approach. (**A**) Accuracy (wAUC) comparison between RBscore and the SVM approach on 14 RBP datasets and 11 DBP datasets, accuracies are shown as heat map with rainbow colors from blue to red as low to high. (**B**) Maximum accuracy (wAUC) variation resulted from distance cutoff in binding site definition. RBscore (red) is more stable in accuracy than the SVM approach (blue).

As shown in Figure 1A and Supplementary Table S3, although the SVM approach achieves a much higher accuracy, 0.947 wAUC, on the training set than RBscore, 0.886, it drops significantly to 0.719 in contrast to the stable accuracy of 0.867 for RBscore in test set. Consistently, tAUC and mAUC also demonstrate a stable high accuracy for RBscore on all other datasets no matter what distance cutoff is used to define the NA binding sites. Besides, Figure 1B also illustrates that RBscore is less likely to be subject to accuracy fluctuation with distance cutoff compared with the SVM approach. Its accuracy variation is less than that of the SVM approach on the majority of the datasets. This implies that RBscore is stable in its predictive capability regardless of dataset and distance cutoff used in binding site definition.

Furthermore, RBscore exhibits high prediction accuracies consistently on all 11 DBP test sets with ∼0.90 wAUC, compared with <0.80 wAUC for the SVM approach (see Table 1 and Figure 1). According to the results, the conclusion also holds true as the distance cutoff used to define binding sites change or assessed with other accuracy criteria. Unexpectedly, RBscore achieves even higher accuracy on DBP than on RBP. This demonstrates that although RBscore is trained with cross-validation on RBP, it can capture the key features of all NA-binding residues including DBP. Also, we have an indication that proteins bind to both DNA and RNA following the same rules of recognition or employ the same driving force, such as electrostatics potential and residue accessibility.

**Table 1.** Accuracy tests on 25 datasets

	RBscore			SVM			aaRNA			RNABindRPlus			BindN+_RNA			BindN+_DNA		
Cutoff = 3.5 Å	wAUC	mAUC	tAUC	wAUC	mAUC	tAUC	wAUC	mAUC	tAUC	wAUC	mAUC	tAUC	wAUC	mAUC	tAUC	wAUC	mAUC	tAUC
BindN_R107	0.850	0.847	0.866	0.878	0.898	0.943	0.827	0.828	0.877	0.907	0.884	0.936	0.897	0.893	0.925	0.761	0.765	0.815
PPRInt_R86	0.857	0.855	0.863	0.884	0.910	0.947	0.835	0.835	0.883	0.918	0.909	0.946	0.871	0.876	0.913	0.771	0.777	0.810
RNABindR_R144	0.860	0.849	0.868	0.849	0.865	0.921	0.828	0.827	0.877	0.894	0.865	0.922	0.819	0.828	0.882	0.737	0.753	0.801
RNABindR_R147	0.860	0.848	0.868	0.849	0.865	0.922	0.828	0.826	0.877	0.894	0.865	0.922	0.819	0.828	0.883	0.737	0.752	0.802
RNABindR_R44	0.862	0.863	0.869	0.810	0.819	0.844	0.817	0.822	0.845	0.763	0.770	0.800	0.784	0.792	0.822	0.754	0.764	0.790
RNABindR_R111	0.898	0.869	0.867	0.789	0.787	0.839	0.849	0.825	0.842	0.762	0.739	0.740	0.768	0.755	0.780	0.748	0.733	0.767
meta2_R44	0.862	0.863	0.869	0.810	0.819	0.844	0.817	0.822	0.845	0.763	0.770	0.800	0.784	0.792	0.822	0.754	0.764	0.790
aaRNA_R67	0.857	0.857	0.874	0.753	0.777	0.815	0.814	0.812	0.846	0.757	0.755	0.776	0.764	0.777	0.810	0.738	0.744	0.783
aaRNA_R141	0.875	0.858	0.836	0.814	0.811	0.848	0.834	0.820	0.835	0.846	0.836	0.854	0.780	0.781	0.792	0.736	0.739	0.731
aaRNA_R205	0.877	0.864	0.867	0.836	0.854	0.911	0.841	0.837	0.878	0.847	0.834	0.881	0.795	0.808	0.853	0.748	0.759	0.792
RBscore_R130	0.886	0.870	0.864	0.947	0.947	0.969	0.838	0.832	0.877	0.828	0.822	0.871	0.806	0.821	0.867	0.759	0.765	0.801
RBscore_R117	0.867	0.855	0.843	0.719	0.723	0.774	0.829	0.820	0.852	0.798	0.789	0.826	0.743	0.747	0.783	0.723	0.728	0.754
Sungwook_R267	0.865	0.848	0.837	0.874	0.865	0.886	0.830	0.811	0.824	0.808	0.796	0.808	0.797	0.783	0.815	0.744	0.741	0.742
Sungwook_R727	0.867	0.857	0.881	0.893	0.894	0.935	0.839	0.833	0.879	0.827	0.820	0.869	0.821	0.822	0.874	0.768	0.773	0.824
BindN_D62	0.909	0.897	0.864	0.776	0.787	0.787	0.881	0.875	0.862	0.787	0.788	0.786	0.822	0.826	0.838	0.944	0.938	0.944
Shandar_D140	0.900	0.893	0.878	0.765	0.772	0.780	0.852	0.855	0.857	0.778	0.778	0.760	0.820	0.821	0.832	0.834	0.855	0.852
Susan_D56	0.918	0.912	0.890	0.766	0.776	0.775	0.872	0.870	0.867	0.766	0.774	0.759	0.808	0.814	0.823	0.843	0.872	0.860
DBindR_D374	0.895	0.887	0.874	0.748	0.757	0.780	0.856	0.856	0.862	0.772	0.777	0.768	0.795	0.807	0.823	0.813	0.840	0.843
DISPLAR_D428	0.894	0.885	0.869	0.757	0.764	0.774	0.854	0.853	0.860	0.775	0.780	0.771	0.803	0.812	0.825	0.824	0.846	0.847
DNABINDPROT_D54	0.920	0.903	0.867	0.723	0.736	0.756	0.864	0.851	0.853	0.754	0.745	0.700	0.786	0.790	0.793	0.807	0.827	0.818
PreDNA_D224	0.896	0.889	0.873	0.748	0.747	0.759	0.857	0.857	0.859	0.764	0.768	0.758	0.794	0.800	0.812	0.802	0.819	0.823
RBscore_D381	0.895	0.884	0.875	0.748	0.742	0.758	0.852	0.844	0.854	0.761	0.764	0.768	0.797	0.795	0.806	0.796	0.805	0.810
metaDBSite_D232	0.898	0.889	0.872	0.753	0.744	0.761	0.858	0.856	0.858	0.769	0.769	0.763	0.798	0.799	0.812	0.804	0.818	0.821
metaDBSite_D316	0.898	0.885	0.878	0.755	0.756	0.772	0.853	0.851	0.859	0.770	0.770	0.769	0.799	0.802	0.818	0.809	0.829	0.834
SDCPred_D159	0.902	0.896	0.880	0.760	0.769	0.773	0.854	0.857	0.856	0.772	0.772	0.753	0.812	0.814	0.823	0.827	0.848	0.843

Datasets after BindN_D62 are DBP datasets. RBscore_R130 is the training set of RBscore and the SVM approach. See Materials and Methods for descriptions of the dataset.

### Contributions of the three features and neighboring network

**Figure 2.**
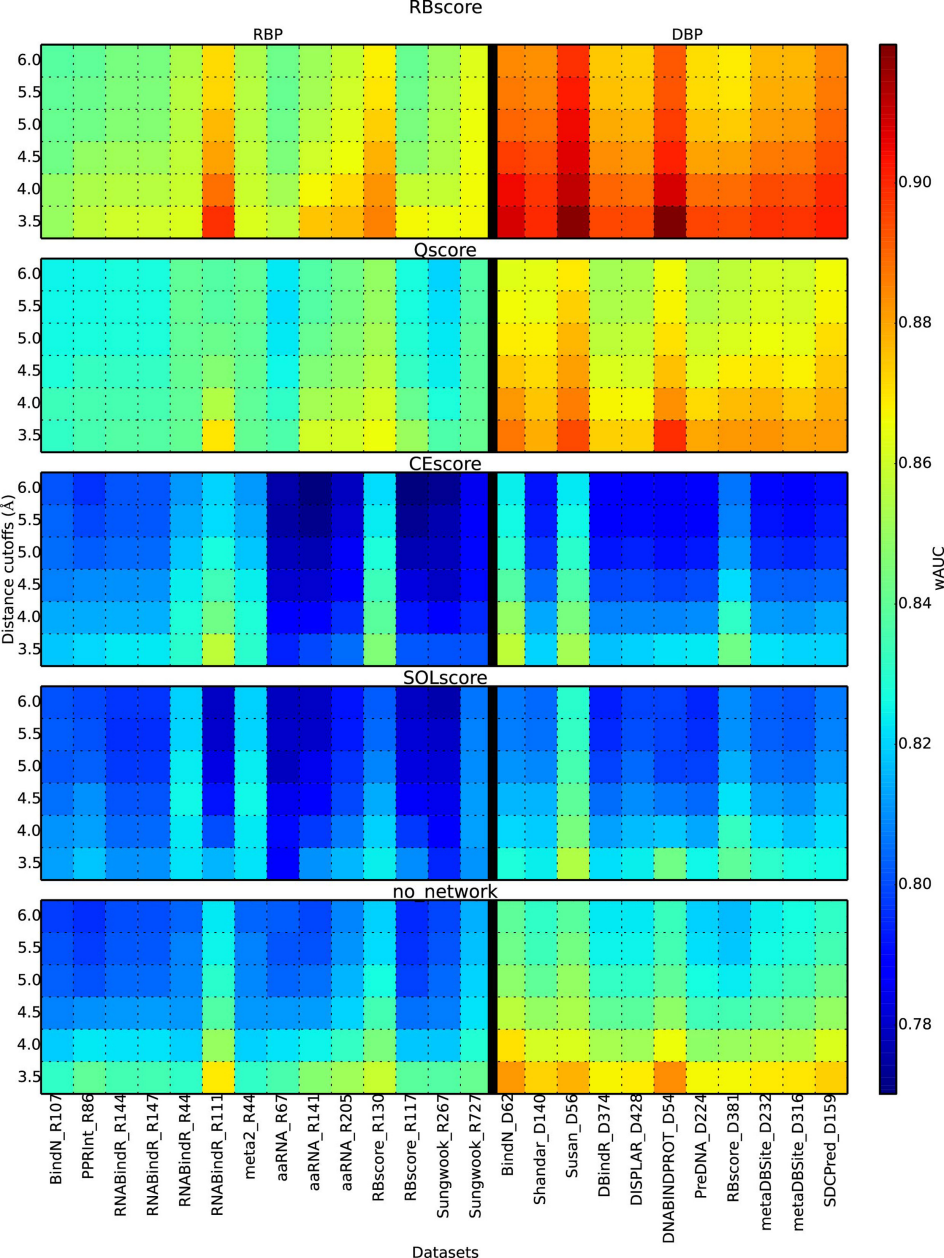
Feature and network integrations improve the prediction. Accuracy (wAUC) comparison between RBscore and single feature based predictions. Qscore, CEscore and SOLscore are predictions based on electrostatics, sequence CE and solvation energy respectively. no_network is prediction without considering the residue neighboring network. RBscore shows systematic improvement over other predictions.

As the three features (Electrostatics, CE and Solvation energy, see Materials and Methods for detail) used in RBscore stand for different aspects of NA-binding residues, Figure 2 and Supplementary Table S3 illustrate that there is no overlap amongst them and that each of these features improves the prediction accuracy. RBscore is consistently better than predictions with single feature alone. And such improvements hold true when tested on every dataset.

The neighboring networks in RBscore include two parts: (i) the spatial neighborhood (based on surface continuity, as described in Materials and Methods) for residues forming a binding interface as a continuous patch on the protein surface, and (ii) the sequential neighborhood that reflects the covalent and local non-bonded contacts. With these two neighboring networks who linearly combine the features, the prediction improves stably on every dataset (illustrated in Supplementary Table S3 and Figure 2) with a wAUC increase 0.02–0.03.

### Comparison with other programs

**Figure 3.**
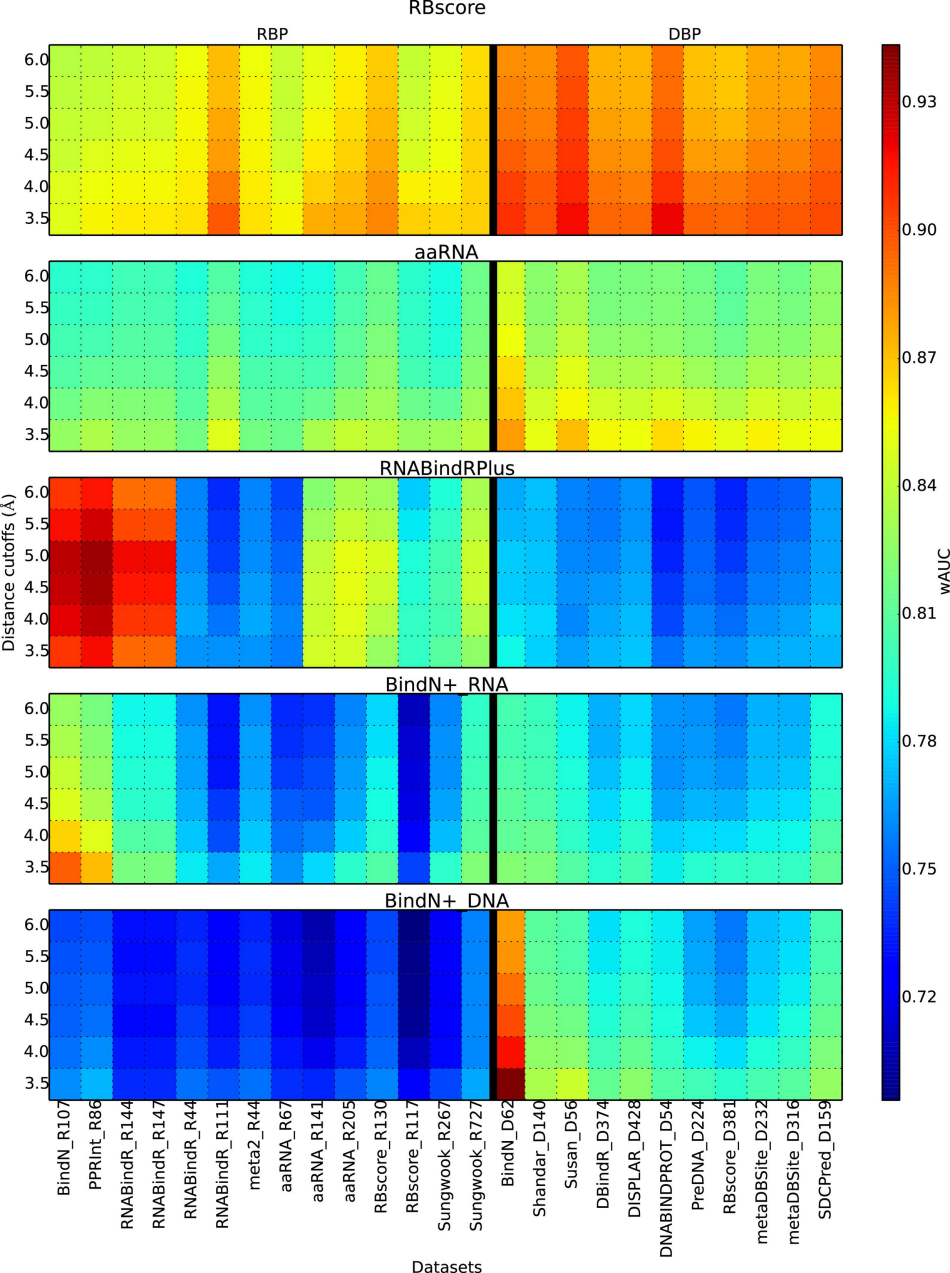
Accuracy comparison among the best prediction programs. BindN+_RNA and BindN+_DNA show the two types of prediction models in BindN+. Both RBscore and aaRNA show stable accuracy on all datasets, while RBscore is systematically better than aaRNA.

For comparisons, nine currently available web servers (BindN (12), BindN+ (5), PPRInt (16), KYG (54), RNABindRPlus (6), DISPLAR (24), DBS-Pred (21)), DBS-PSSM (55) and (19) for predicting RNA/DNA binding were tested together with RBscore and the SVM approach on 25 different datasets (see Supplementary Table S3 for detailed results). The top three programs BindN+, RNABindRPlus and aaRNA are compared in Figure 3.

A priori, it is not surprising that DBP and RBP may adopt similar driving features in binding and the prediction programs can achieve the two simultaneously. However, such a prediction achievement is not reached by most predictors, except for RBscore and aaRNA (see Figure 3). Machine-learning methods can demonstrate strong advantages in interpolation but not in extrapolation, the prediction power on DBP may be limited.

The results for BindN and KYG show stable but low prediction accuracies, 0.68–0.72 and 0.73–0.77. BindN+ and PPRInt present high accuracy in terms of total AUC, but are very unstable in different datasets. wAUC ranges are 0.76–0.89 and 0.70–0.85, while mAUC also shows the same trends. As BindN+ employ two SVM models to predict RNA and DNA binding sites, each model performs better on its respecting type of proteins. RNABindRPlus, that integrates machine learning and homologous search strategies in RNA binding site prediction, shows good accuracies of tAUC on some of the RBP datasets, but is less accurate in terms of wAUC and in DBP datasets. In brief, relative to all these comparisons, RBscore achieves wAUC >0.85 (3.5 Å distance cutoff) on all types of datasets with stable accuracies.

As different programs may use different distance cutoffs to define RNA binding sites, assessments with different cutoffs from 3.5 Å to 6 Å were also carried out and similar conclusions could be drawn. We found that the programs normally favor the implemented distance cutoffs, but the accuracy variations are less than the differences between different datasets. Generally, wAUC accuracies for RBscore are still in the range >0.83. The machine-learning-based methods (5,16) have similar philosophy and accuracy distribution as our SVM approach. This may result from (i) the bias introduced by cross-validation in pattern recognition (56) (see Supplementary Note 3 for discussion); (ii) some sequence-based predictors include datasets without removing structural homology, leading to the overestimation in accuracy (see Supplementary Note 4 for discussion). Collectively, these results demonstrate that RBscore achieves both accuracy and stability in accuracy that cannot be achieved by other currently available programs. Full comparison could be found in Supplementary Figure S3.

### Energy funnel

**Figure 4.**
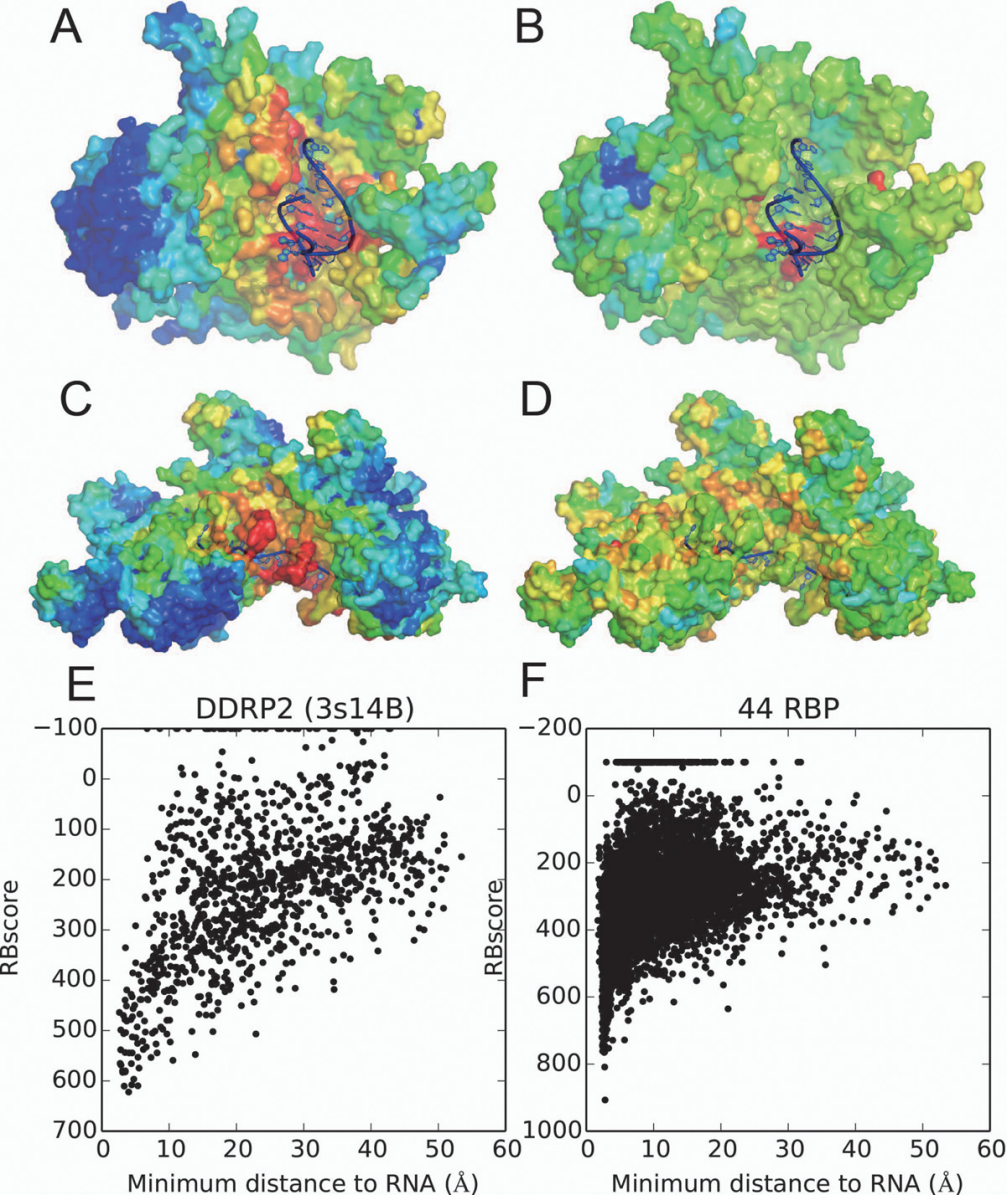
Energy funnel comparison on protein surface. (**A**) RBscore mapped on DNA-directed RNA polymerase II (DDRP2) subunit RPB2, PDB id 3S14 chain B (not in the training set) with rainbow color. (**B**) SVM score mapped on DDRP2 protein in the same way. (**C**) RBscore mapped on DBP recA, PDB id 3cmw chain A (not in the training set) with rainbow color. (**D**) SVM score mapped on recA protein in the same way. (**E**) The relation between distance to RNA and RBscore clearly shows an energy funnel-like pattern of DDRP2 protein. (**F**) A distribution between distance to RNA and RBscore on 44 RBP.

As a result of the neighboring network based scoring that can capture the neighboring environment of a residue in a network approach, RBscore is normally continuous on the protein surface and varies with the minimum distance from the protein residue to NA. Compared with SVM scores mapped on the protein surface (Figure 4A and C), RBscore shows a hierarchical scoring on the protein surface. The approximate continuity of RBscore on the protein surface results from two points: (i) the neighboring network approach implemented in RBscore considers not only the features of the residue but also the neighboring environment; and (ii) unlike the machine-learning classifiers that try to cluster positive and negative samples around two fixed values (for example −1 and 1), RBscore does not set a limit in scoring but attempts to represent the NA binding probabilities. Thus, RBscore represents adequately the binding probability of NA and the energy funnel on protein surface. When we compare RBscore with the minimum distance from a residue to NA (Figure 4E), we find that the residues closer to NA normally have better RBscore and the distribution is similar to an energy funnel that has been described in molecular docking. Although mixing all the proteins together for comparison is unreasonable, Figure 4F still shows the trends of an energy funnel when 44 RBPs were mixed together. Additionally, we measured the correlation coefficient between RBscore and the minimum distance from a residue to RNA/DNA for residues around the binding region. The average values of these correlation coefficients of all the test sets are listed in Supplementary Table S4. As all the average correlation coefficients of the datasets are around 0.5, we conclude that RBscore is positively related to the distance from a residue to RNA/DNA. If RBscore is related to the binding energy funnel on the protein surface, it can be correlated with the distance from a residue to the core of the binding region of the interface. The core of binding region is hard to define, but the minimum distance from a residue to RNA/DNA can monitor (at least partly) the residue affinity to RNA/DNA. The positive correlation coefficients around 0.5 support that RBscore is related to the binding energy funnel on protein surface. According to the energy funnel colored by RBscore on protein surface, we guess that the protein–NA binding process could follow dynamic process: NA may first bind to sub-optimal locations and slide alongside down the energy funnel. Simultaneously, we hope RBscore could help NA–protein docking by avoiding the search of all degrees of freedom.

### Estimate the RNA binding site number from sequence

Unlike a binary prediction (binding or non-binding), RBscore displays the probability of NA binding and illustrates how far away a residue is from the NA binding region. It can therefore be valuable for estimating the number of NA-binding residues in a protein. We found that the number of NA-binding residues of a protein is highly correlated to the proportion of six types of residues in the protein sequence (see Supplementary Note 5 and Supplementary Figure S4). Some other residue types are also related to RNA/DNA binding, but their proportions do not have positive correlation with the number of binding sites. These six residue types are Arg, Asp, Gly, His, Lys and Thr. They are similar to the important interface residues for RNA binding found in previous analysis (57). Interestingly, three of them (R, G, K) belong to the disorder-promoting amino acid types and the three other belong to the ambivalent class (H, T, D) (58). Even if RNA-binding residues are defined by different distance cutoffs or tested in different datasets, the Pearson correlation efficiency between the ratios of the six residues and ratios of RNA-binding residues is always around 0.8. Thus, the number of NA binding sites can be roughly estimated according to the proportion of these six residue types, a detailed example can be found in Supplementary Figure S4.

### Prediction based on homologous structures

**Figure 5.**
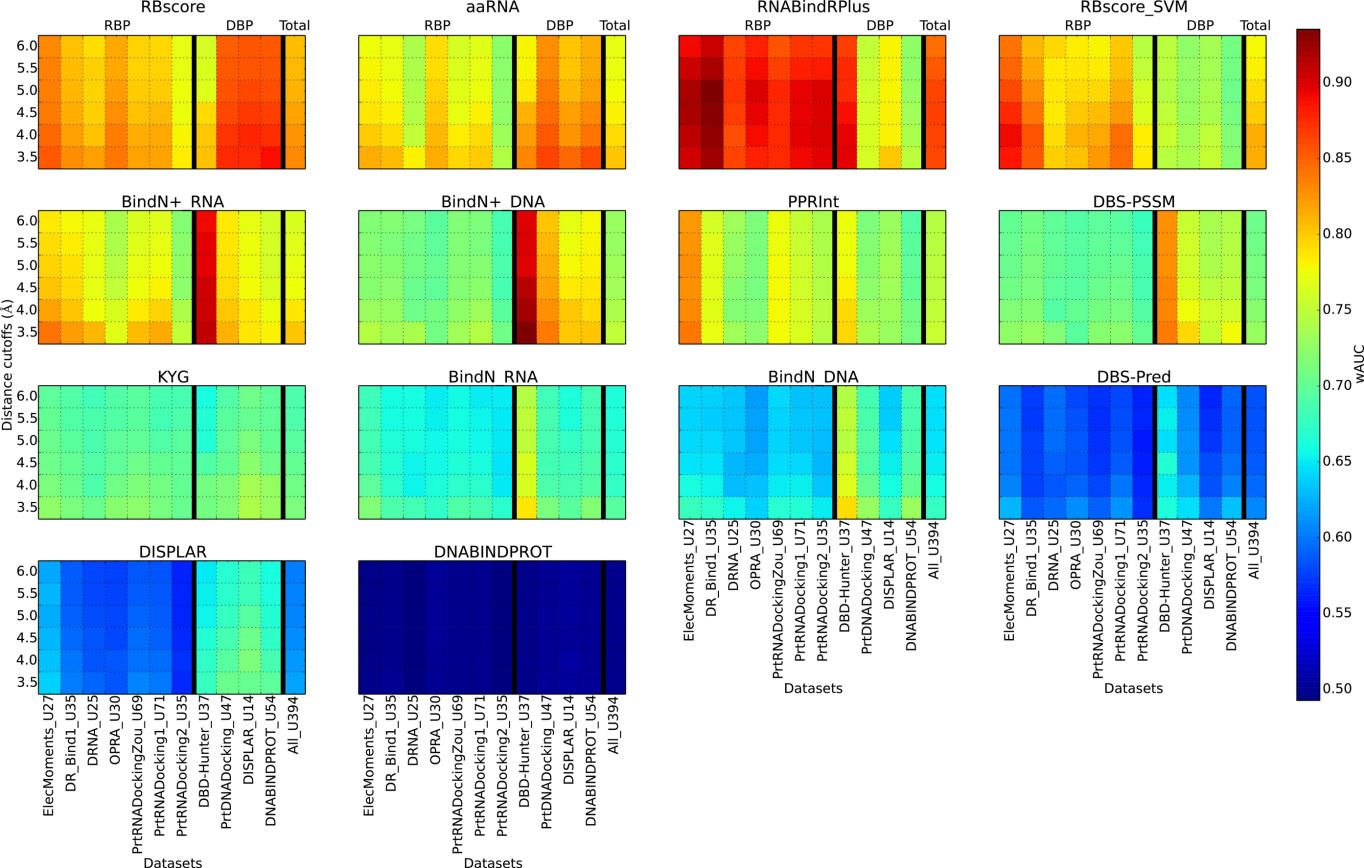
Prediction accuracy assessment based on non-native structures. RBscore, aaRNA, KYG, DISPLAR and DNABINDPROT are structure-based predictors, and other predictions are based on sequence. Accuracies of the programs on different datasets (seven RBP datasets and four DBP datasets) determined by binding sites defined by different distance cutoffs (from 3.5 to 6.0 Å) are plotted as heat maps.

In a real-world case, since the starting structure influences the prediction accuracy, prediction should be based on the unbound state structure rather than on the bound state. Therefore, we tested RBscore together with other programs on 11 unbound protein datasets, including seven RBP datasets and four DBP datasets. Each protein in the datasets had a corresponding bound state structure. Structures used in prediction were modeled by homology modeling according to the unbound state structure as the template and predicted binding sites are compared with the observed binding sites in the bound state. The results are plotted in Figure 5, while the relationships between the predicted structure model quality and binding site prediction accuracy are plotted in Supplementary Figure S5. Similarly to the test shown in Figure 3, RBscore and aaRNA demonstrate stable high accuracy on all the datasets, while RNABindRPlus exert highest accuracy in all RBP datasets but accuracy drops on three DBP datasets. Such high accuracy on RBP datasets may be attributed to the homologous search approach (HomPRIP) integrated in RNABindRPlus. Although tested with limited number of proteins in unbound test, RBscore still achieves one of the best predictions. And this shows that RBscore predictions are tolerant to structural variation or noise.

### Web server description

The prediction method is available as a web server at http://ahsoka.u-strasbg.fr/rbscore/. Both RBscore and SVM approaches are carried out when a protein structure is available as input in the PDB format. Scores predicted by RBscore with different features are plotted on the protein structure and illustrated with JSmol (59). Besides, the electrostatics potential is also mapped onto the protein surface as well as the Shannon CE, similarly to PatchFinderPlus (7,39) and Consurf (9,60). If only the protein sequence is available, the prediction is based only on the SVM approach and only the prediction score and binary prediction of RNA binding sites are given as results. The results of the prediction are returned by email and by web page updates. All the datasets used in this work are also available on the website.

## DISCUSSION

RBscore is first built on three main physicochemical and evolutionary features that are subsequently integrated into a neighboring network as a linear combination. The score, thus, directly transforms the key features by weighing factors into NA binding probability without the complicated process of machine learning or database search. As NA-binding residues on a protein normally occur as patches, the neighboring network that considers both structural and sequential neighborhoods not only helps to describe the relations between residue neighbors of NA binding patches but also makes RBscore continuous on the protein surface. As a probability score, RBscore avoids unnecessary comparisons between residues of different proteins and uses wAUC and mAUC as criteria to achieve a better accuracy for each predicted protein. Furthermore, this work revealed a strong linear correlation between sequence composition (R, G, K, H, T, D) and number of binding sites. This correlation can be used to estimate roughly the size of the NA binding region given the sequence.

Surprisingly, RBscore achieves high accuracies on DBP although it was first developed for RBP, which underscores that DBP and RBP incorporate the same general rules responsible for binding NA. Interestingly, when RBscore is mapped onto protein structures, we found that it displays energy funnel patterns. Further, the 2D distribution patterns of the scores are similar to the energy funnel plots between ligand RMSD in protein–protein docking and energy in protein folding. When the energy funnels of protein–protein docking, protein folding and RBscore are compared together in Supplementary Figure S6, similar patterns are displayed illustrating the energy funnel like patterns followed by RBscore. Therefore, one can imagine the NA–protein binding process as following dynamic process with NA first binding to sub-optimal locations and then sliding alongside down the energy funnel. This dynamic process, constrained by each residue environment, can be partly described by the proposed residue neighboring network incorporated in RBscore.

Compared to numerous programs on various datasets with different criteria, we found that RBscore has consistently wAUC >0.83 on all datasets regardless of the protein types (DBP/RBP) or distance cutoff used to define binding sites, a result that cannot be achieved by other currently available programs. Still, one can find certain machine-learning methods performing better on some datasets.

The general features used in RBscore cannot capture all the detailed binding properties of all proteins unless overtrained. Compared to many machine-learning approaches that employ the PSSM facility, the number of parameters in RBscore is not large. Indeed, the number of parameters in the machine-learning prediction models would always be larger than the number of input vector, which may include *n*×20, where *n* is the window length (61). For instance, the reference SVM approach of 11-mer window length has a 220-column input vector. Normally, parameters in machine-learning models are much larger than this: aaRNA has >668 parameters and DISPLAR includes 195 840 parameters. Compared with these numbers of parameters, RBscore of 104 weighing factors is relatively small. And the stable accuracy on all types of datasets regardless of distance cutoff difference implies that it is less likely to be overtrained than others.

RBscore is a general score to predict NA binding that presents both advantages and limitations. First, it is not able to assess or predict the type of the input protein and distinguish whether it is a DBP or an RBP, simultaneously with the prediction of binding sites. Indeed, by construction, it is not meant to distinguish between the binding regions specific for different types of ligands (RNA, DNA, small molecules or ions). For example, the ANP binding region and the RNA binding region on the DDX protein (Supplementary Note 6 and Supplementary Figure S7) are both scored high and the two different binding types cannot be reflected in the score. Second, RBscore only detects the general interface for NA binding or ligand binding and does not carry along either the NA sequence specificity of the binding site or the states of NA (single-strand or double-stranded). These apparent drawbacks have positive sides too. It is now clear that many proteins do not contain canonical RNA binding motifs (like RRM, KH or Znf domains (62)). Furthermore, some proteins may display non-specific (or promiscuous) interactions with RNA (as in Polycomb complex (63)) or ‘cryptic’ affinities for RNAs (moonlighting proteins as the enzyme aconitase or other metabolic enzymes (64,65)). RBscore does not need a knowledge of canonical or non-canonical binding motifs since it attempts to find the residues that have favorable features for RNA/DNA binding and combine these residues in a network, independent of the presence of canonical or non-canonical RNA binding motifs. For RBscore, the driving force is the main determinant for protein–RNA binding sites rather than the specificity and, thus, a promiscuous or cryptic binding site can still be detected. Finally, despite the observation that the higher scored residues in the center of a binding region are normally more specific than other residues, the validation of the specificity prediction is still to be clarified. Generally speaking, this problem is not solved and is also germane for other programs.

In summary, RBscore relates through structural networks the physicochemical and evolutionary features to NA binding, shows the presence of an energy funnel for protein–NA binding and achieves high and stable prediction accuracies.

## AVAILABILITY

RBscore is available as web server to non-commercial users on http://ahsoka.u-strasbg.fr/rbscore/.

## SUPPLEMENTARY DATA

Supplementary Data are available at NAR Online.

SUPPLEMENTARY DATA
