# Supplementary material for: Prediction of nucleic acid binding probability in proteins: a neighboring residue network based score
Source: Nucleic Acids Res. 2015 May 4;43(11):5340–51. doi: 10.1093/nar/gkv446 (PMC4477668; doi:10.1093/nar/gkv446)
Supplement: SUPPLEMENTARY DATA [file supp_43_11_5340__index.html]

Prediction of nucleic acid binding probability in proteins: a neighboring residue network based score — Prediction of nucleic acid binding probability in proteins: a neighboring residue network based score — SUPPLEMENTARY DATA 

# Prediction of nucleic acid binding probability in proteins: a neighboring residue network based score

## SUPPLEMENTARY DATA

**Files in this Data Supplement:**

- SUPPLEMENTARY DATA
- SUPPLEMENTARY DATA
