# Supplementary material for: Prediction of nucleic acid binding probability in proteins: a neighboring residue network based score
Source: Nucleic Acids Res. 2015 May 4;43(11):5340–51. doi: 10.1093/nar/gkv446 (PMC4477668; doi:10.1093/nar/gkv446)
Supplement: SUPPLEMENTARY DATA [file supp_gkv446_nar-00801-h-2015-File008.docx]

**Prediction of nucleic acid binding probability in proteins: a neighboring residue network based score**

Zhichao Miao and Eric Westhof*

Architecture et Réactivité de l'ARN, Université de Strasbourg, Institut de biologie moléculaire et cellulaire du CNRS, 15 Rue Descartes 67000 Strasbourg France

* To whom correspondence should be addressed. Tel: +33388417046; Fax:+33388602218; Email: [e.westhof@ibmc-cnrs.unistra.fr](mailto:e.westhof@ibmc-cnrs.unistra.fr)

Present Address: Eric Westhof, Architecture et Réactivité de l'ARN, Université de Strasbourg, Institut de biologie moléculaire et cellulaire du CNRS, 15 Rue Descartes 67000 Strasbourg France

**Note 1.** Biased assessment of binding site prediction accuracy based on total AUC.

Current prediction methods for NA binding residues normally compare all residues in all proteins together to measure the area under the ROC curve (AUC) for assessment. However, different NA binding proteins may have different affinity to NA ([Yang et al, 2013](#_ENREF_25)) and, thus, different proteins should not be treated similarly. For binding sites prediction, the main aim is to locate the key binding region rather than all the details of binding sites, and we only need to discriminate binding residues against non-binding residues in the same protein. Thus, the current measurement, that uses total AUC to compare all residues from all proteins together, biases the accuracy by overemphasizing comparisons between residues of different proteins. A simple illustration is shown in **Fig S8**. Therefore, we suggest, here, to use as criteria for accuracy, the weighted averaged AUC and averaged AUC values (wAUC and mAUC described in methods), with the total AUC (tAUC, current measurement) used as a reference. wAUC is the weighted average of all AUC of proteins in a dataset, while mAUC is the mean of all AUC of proteins in a dataset.

As a simple example, we can find the accuracy of SVM on RBscore_R117 dataset (in **Table 1**). tAUC is 0.83 while wAUC and mAUC are 0.78 and 0.78 respectively. tAUC seems higher in accuracy than wAUC and mAUC, because it overemphasizes the comparison between residues of different proteins. Contrarily, with a low tAUC, a prediction on each protein can still be very accurate as long as wAUC and mAUC are high.

**Note 2.** The influence on prediction accuracy in dependence with the distance cutoff used for defining binding sites.

In previous studies ([Kumar et al, 2008](#_ENREF_10); [Ma et al, 2011](#_ENREF_14); [Terribilini et al, 2006](#_ENREF_16); [Terribilini et al, 2007](#_ENREF_17); [Wang et al, 2010](#_ENREF_23)), NA binding residues were always defined as residues that have at least one nucleic acid atom in contact within a distance cutoff, while other methods ([Cheng et al, 2008](#_ENREF_5); [Li & Li, 2012](#_ENREF_11); [Liu et al, 2010](#_ENREF_12); [Wang et al, 2011](#_ENREF_21); [Wang et al, 2008](#_ENREF_24)) use program ENTANGLE ([Allers & Shamoo, 2001](#_ENREF_2)) to define binding sites. The distance cutoffs range from 3.5 ([Wang et al, 2010](#_ENREF_23)) to 7Å ([Kim et al, 2006](#_ENREF_8)). Define binding sites with a long distance cutoff but without considering the accessible surface area change (ΔASA) can lead to a situation where a totally buried residue is defined as a binding site. Therefore, ΔASA could be an additional parameter for distance cutoff in defining NA binding sites.

Nevertheless, a definition of nucleic acid binding sites is always limited by our knowledge of the assembled structures adopted as we can deduce from a PDB file. Spatial proximity in crystal structure does not always necessary mean binding. A recent example is the Cascade complex ([Zhao et al, 2014](#_ENREF_26)) (see **Fig S9** for RBscore prediction). As a RNA-guided complex, the Cascade complex cannot form directly a complex with RNA. But the proteins are structurally very close. This remind us of the situation where two molecules close to each other in a complex structure may not bind unless brought together by a third partner. Therefore, a definition based on the PDB assembled structures is also imperfect for nucleic binding sites.

According to the results obtained, the programs favor the distance cutoffs used as thresholds. In principle, these methods are also similar to our SVM approach since most of them are machine learning based strategies, despite their utilization of features generated by sequence itself or by structure and what kind of classifier used in the prediction. As an example, BindN+ ([Wang et al, 2010](#_ENREF_23)) uses 3.5Å as distance cutoff, while RNABindRPlus uses 5Å. We find that BindN+ has higher performance when using 3.5Å to define the binding sites compared with RNABindRPlus. Thus, hierarchical distance cutoffs are used in our tests to fully capture the differences between programs.

**Note 3.** The problem of data set bias introduced by cross-validation.

Both machine learning and database search approaches are pattern recognition methods and most of the previous programs were verified by cross-validation. However, there are two assumptions necessary for cross-validation: 1) the number of folds is not too small; 2) the data result from random sampling. Recent work ([Braga-Neto et al, 2014](#_ENREF_4)) shows that predictions without these assumptions result in strong bias. Nevertheless, these assumptions were not validated before applying cross-validation, since only less than 1/1000th proteins of known sequences have experimental structures available ([Moult, 2008](#_ENREF_15)) and RNA related structures take up less than 6% of the structure database ([Berman et al, 2000](#_ENREF_3)). Besides, the set of solved structures is not the result of random sampling since it is strongly dependent on the ease of purification and of crystallization. This could explain the low performance of SVM on test set R117 as well as with other datasets. Normally, a SVM based strategy would result in tens of thousands of support vectors in the prediction model. And it may have a memory of the training set rather than only the key features of nucleic acid binding. In contrast, RBscore includes only 104 parameters in linear combination of the feature values to capture the key features of nucleic acid binding. To further exclude the possibility of data set bias, we collected 23 different data sets from previous studies, together with the three datasets in this work, to make a comprehensive test compared with ten of the currently available data sets (**Table S3**).

**Note 4.** Homologous proteins can have low sequence identity but similar structure.

Sequence identity less than 25% or 30% is normally used as a criterion to remove redundancy due to homology between proteins in a dataset. However, this is not necessarily enough in the case of binding site prediction. For example, both 1dfu chain P and 1feu chain A are 50s ribosomal protein L25, who fold into identical structures of RMSD 1.37Å as illustrated in **Fig S10A**. But the two structures only share 16.9% sequence identity. Then, we search homologous sequence with HHblits to build sequence profile. As shown in **Fig S10B**, we can find the sequence profiles are nearly the same. That is to say, a protein can be easily predicted if the other exists in the training set. Such a case is commonly seen. As many prediction programs use PSSM or sequence profile in the prediction, including such homologous proteins in the datasets may lead to bias in accuracy assessment.

**Note 5.** The number of NA binding sites strongly correlates with the proportion of six residue types in sequence.

Six residue types Arg, Asp, Gly, His, Lys and Thr clearly correlate with the number of NA binding sites in proteins. They are similar to the important interface residues for RNA binding found in previous analysis([Treger & Westhof, 2001](#_ENREF_19)). Interestingly, three of them (R, G, K) belong to the disorder-promoting amino acid types and the three other belong to the ambivalent class (H, T, D)([Dunker et al, 2001](#_ENREF_7)). Even if RNA binding residues are defined by different distance cutoffs or tested in different datasets, the Pearson correlation efficiency is always around 0.8 between the ratios of the six residues (N_(RDGHKT)_/N_total_) and ratios of RNA binding residues (N_bind_/N_total_). This implies that these six residues play major physicochemical and structural roles in RNA binding, and we can roughly guess the number of RNA binding residues of a protein based on its sequence. As demonstrated in **Fig S4**, most of the residues fall into the region between two lines of standard deviation. For each protein sequence in prediction, we can measure the ratios of the six residues and calculate the maximum and minimum number of binding sites according to the two standard deviation lines.

**Note 6.** The limitations of RBscore in distinguishing different types of ligand binding.

The general features used in RBscore cannot capture all the detailed binding properties of all proteins unless over-trained. As a general feature based score, RBscore cannot distinguish RNA binding sites from DNA binding sites or ligand binding sites.

A simple case is illustrated in **Fig S7**, the RNA helicase ([Collins et al, 2009](#_ENREF_6)) has a RNA binding region and an ANP binding region. As the ANP binding region is normally considered as a false position in the assessment of RNA binding site prediction, RBscore may not show high accuracy in this case. And the accuracy of RBscore is limited by the definition of binding residues. But both the RNA binding region and ligand binding regions are functional and detecting them is beneficial.

RBscore only detects the general interface for nucleic acid binding or ligand binding and does not carry along the specificity of the binding site. Despite the observation that the higher scored residues in the center of a binding region are normally more specific than other residues, the validation of the specificity prediction is still to be clarified.

**Table S1**. Parameters used in RBscore for linear combination.

Parameters including *C_aa_*, *w_i_*, *w_EC_* and *w_width_* are shown in this table.

|  | C_aa_ | w_ASA_ |  | w_i_ for electrostatics |
| --- | --- | --- | --- | --- |
| ALA | -3.81554 | 0.502776 |  | 1.67995 |
| ARG | 17.0485 | 0.396306 |  | 1.99374 |
| ASP | 15.7662 | 0.473709 |  | 2.04309 |
| ASN | 14.1375 | 0.444789 |  | 1.51543 |
| CYS | 6.36046 | 0.211285 |  | 1.33098 |
| GLU | -12.0531 | 0.705317 |  | 2.56704 |
| GLN | 12.8003 | 0.428371 |  | 1.77241 |
| GLY | 4.40431 | 0.494818 |  | 1.77467 |
| HIS | 0.572952 | 0.725995 |  | 2.0104 |
| ILE | -17.9611 | 0.608614 |  | 0.872696 |
| LEU | -19.8535 | 0.773427 |  | W_EC_ |
| LYS | -2.26554 | 0.528065 |  | 2.35434 |
| MET | 9.55724 | 0.387478 |  | W_width_ |
| PHE | -8.77218 | 0.740378 |  | 0.761398 |
| PRO | -20 | 0.569938 |  |  |
| SER | 15.7383 | 0.542555 |  |  |
| THR | 15.9768 | 0.358455 |  |  |
| TRP | -18.1173 | 0.951399 |  |  |
| TYR | -13.6748 | 0.990504 |  |  |
| VAL | -19.6377 | 0.70941 |  |  |

**Table S2.** 5-fold Cross-validation results of RBscore and SVM approach. Cross-validation of RBscore is based on protein id shuffling while SVM shuffles residues.

| wAUC | SVM CV | RBscore CV |
| --- | --- | --- |
| 1 | 0.829351803 | 0.887439 |
| 2 | 0.843997044 | 0.884068 |
| 3 | 0.829528378 | 0.884947 |
| 4 | 0.832124691 | 0.868688 |
| 5 | 0.844192456 | 0.865606 |

**Table S3.** Accuracy assessments of ten available programs on 25 datasets (RBscore_R130 is the training set of RBscore). Programs are used with default parameters. The programs are: BindN+ ([Wang et al, 2010](#_ENREF_23)) (DNA and RNA models respectively), BindN ([Wang & Brown, 2006](#_ENREF_22)) (DNA and RNA models respectively), PPRInt ([Kumar et al, 2008](#_ENREF_10)) (program only for RBP), KYG ([Kim et al, 2006](#_ENREF_8)) (program only for RBP), RNABindRPlus ([Walia et al, 2014](#_ENREF_20)), DBS-Pred ([Ahmad et al, 2004](#_ENREF_1)) and DISPLAR ([Tjong & Zhou, 2007](#_ENREF_18)). DBS-Pred and DISPLAR only give binary predictions of binding sites. For the assessment when comparing with other methods, positive sites are assigned score 1 and negative sites are assigned score -1. This choice can underestimate the value of these two programs but one can compare them in terms of ACC and PPV. The datasets are described in methods. In the table, wAUC and mAUC show the accuracies of the programs while sAUC, i.e. the standard deviation of AUC, shows the stability of the method. All other values overemphasize the comparison between residues from different proteins and are only used as a reference.

**Table S4.** Average correlation coefficient between RBscore and distance to RNA/DNA on all datasets. Only residues around the binding region, who are within 8Å of the RNA/DNA, are considered.

| Dataset | Average Correlation Coefficient |
| --- | --- |
| BindN_R107 | 0.50648339 |
| PPRInt_R86 | 0.50773075 |
| RNABindR_R144 | 0.50233354 |
| RNABindR_R147 | 0.50285921 |
| RNABindR_R44 | 0.52367447 |
| RNABindR_R111 | 0.47504192 |
| meta2_R44 | 0.52367447 |
| aaRNA_R67 | 0.49834447 |
| aaRNA_R141 | 0.46242268 |
| aaRNA_R205 | 0.50552073 |
| RBscore_R130 | 0.52419936 |
| RBscore_R117 | 0.46359234 |
| Sungwook_R267 | 0.46876882 |
| Sungwook_R727 | 0.53270449 |
| BindN_D62 | 0.55326714 |
| Shandar_D140 | 0.54154783 |
| Susan_D56 | 0.55799966 |
| DBindR_D374 | 0.51232845 |
| DISPLAR_D428 | 0.50982281 |
| DNABINDPROT_D54 | 0.55664962 |
| PreDNA_D224 | 0.50277482 |
| RBscore_D381 | 0.50579534 |
| metaDBSite_D232 | 0.50659927 |
| metaDBSite_D316 | 0.50813298 |
| SDCPred_D159 | 0.54197584 |


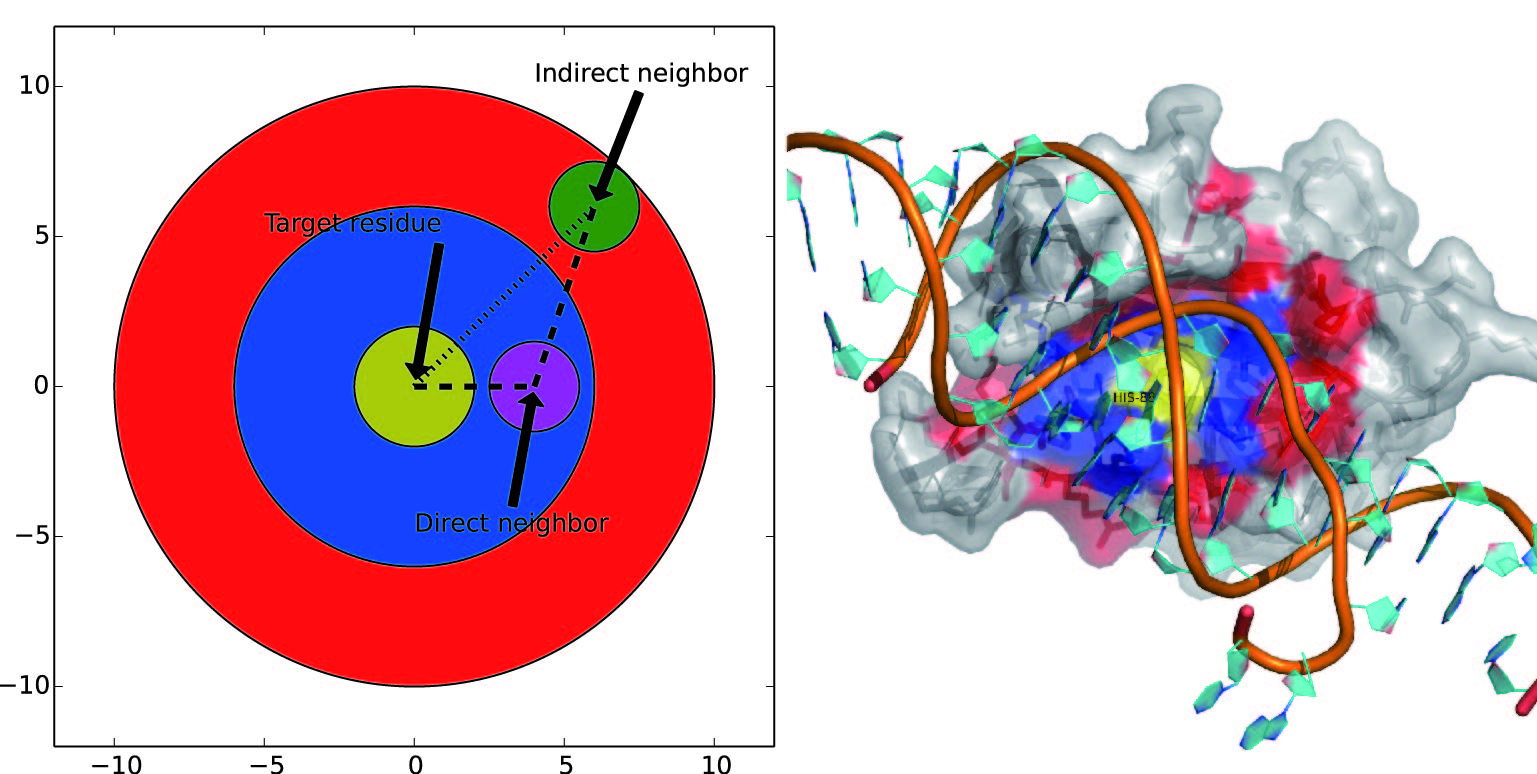


**Figure S1.** Illustration of the neighboring network. For a target residue (yellow), residues that directly interact with it are direct neighbors (blue), residues that do not interact with the target residue but interact with its direct neighbors are assigned as indirect neighbors (red).


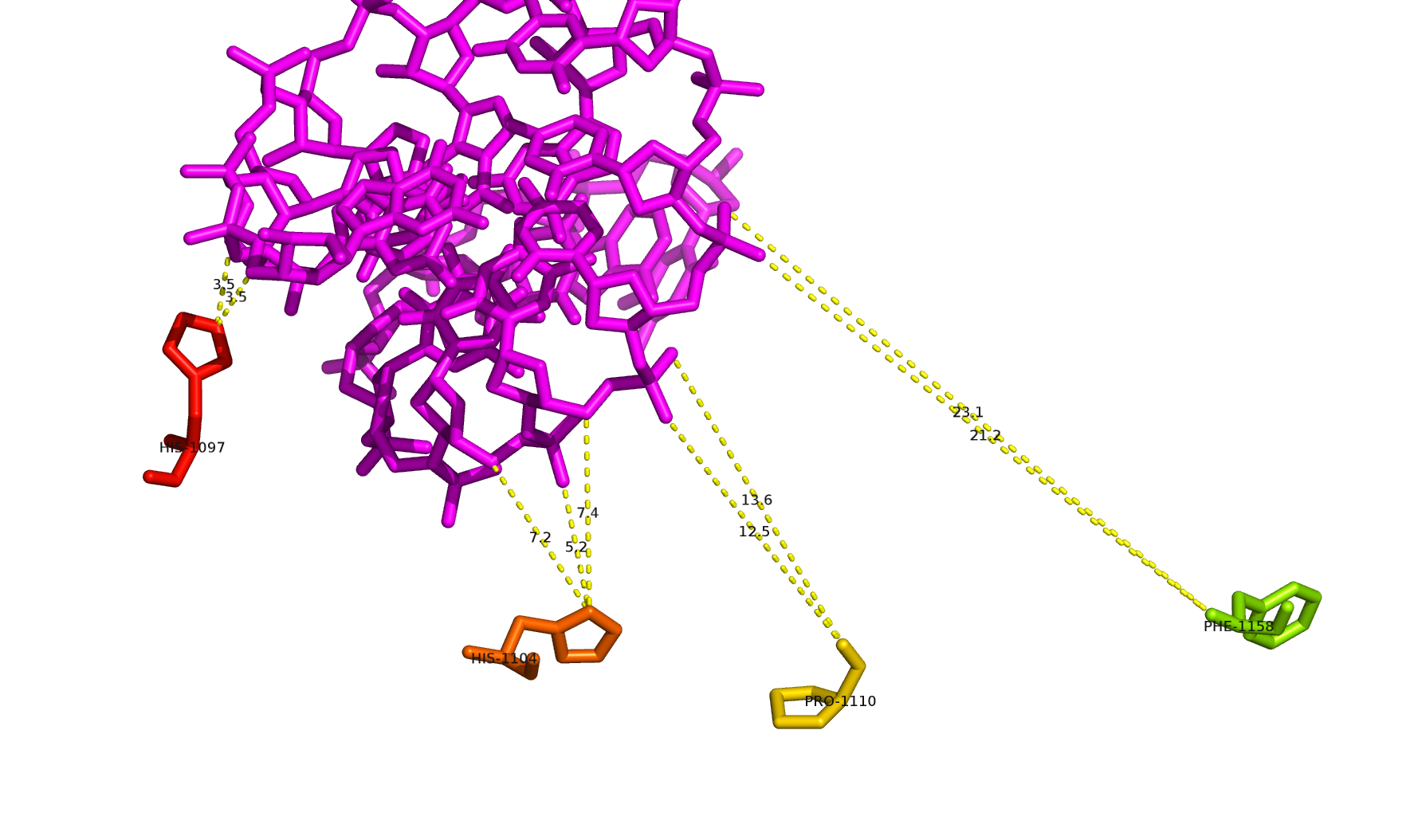


**Figure S2**. Hierarchical scoring of RBscore and related residue distance to RNA resulting energy funnel like pattern. RBscore of the residues (in PDB 3s14 chain B) are colored according to red to green color scheme. Minimum distances from the residues to RNA are marked. Residues further away from RNA have worse RBscore. The four residues form part of the energy funnel on the protein surface.

**
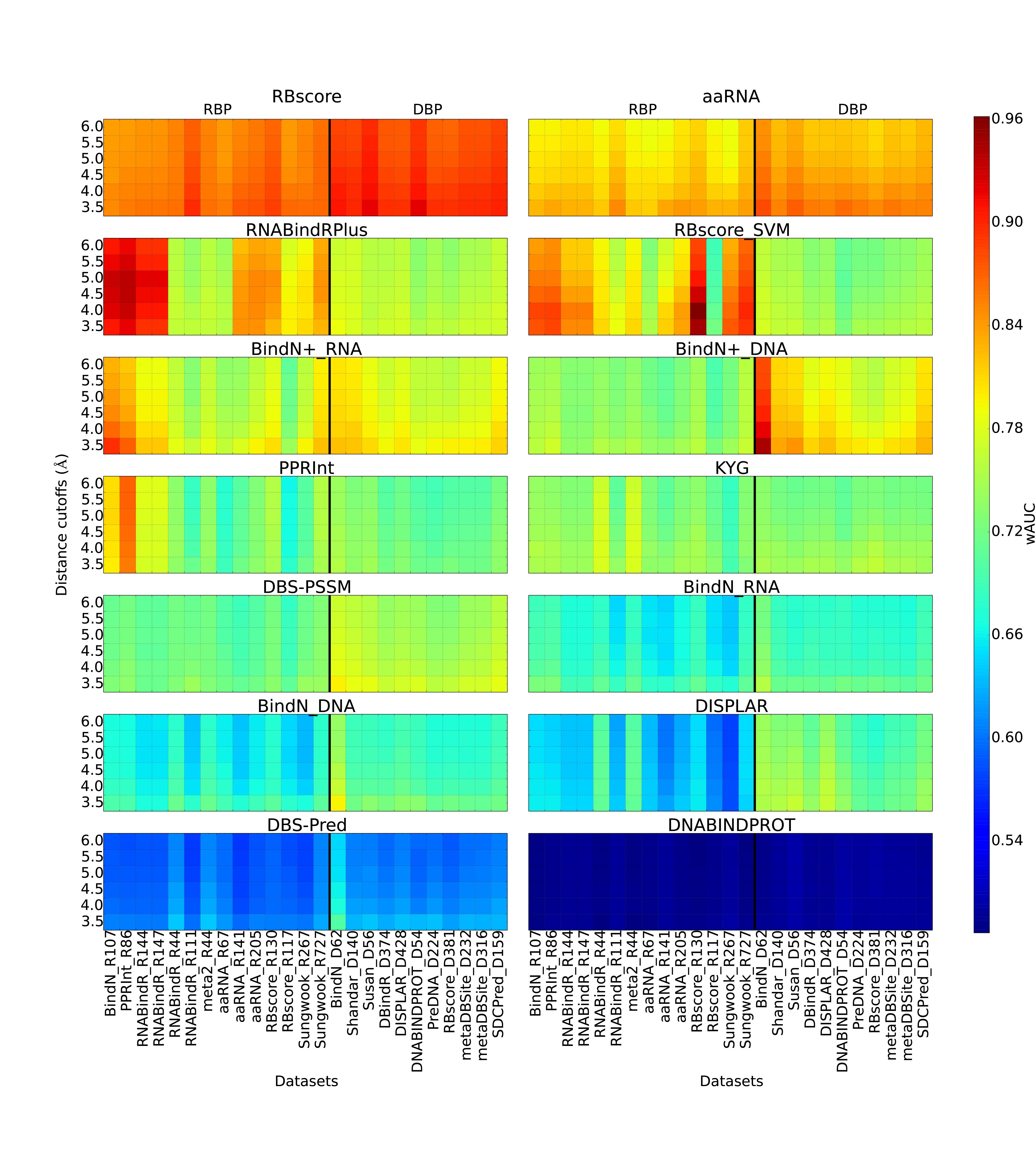
**

**Figure S3.** Accuracy comparison of all the programs.


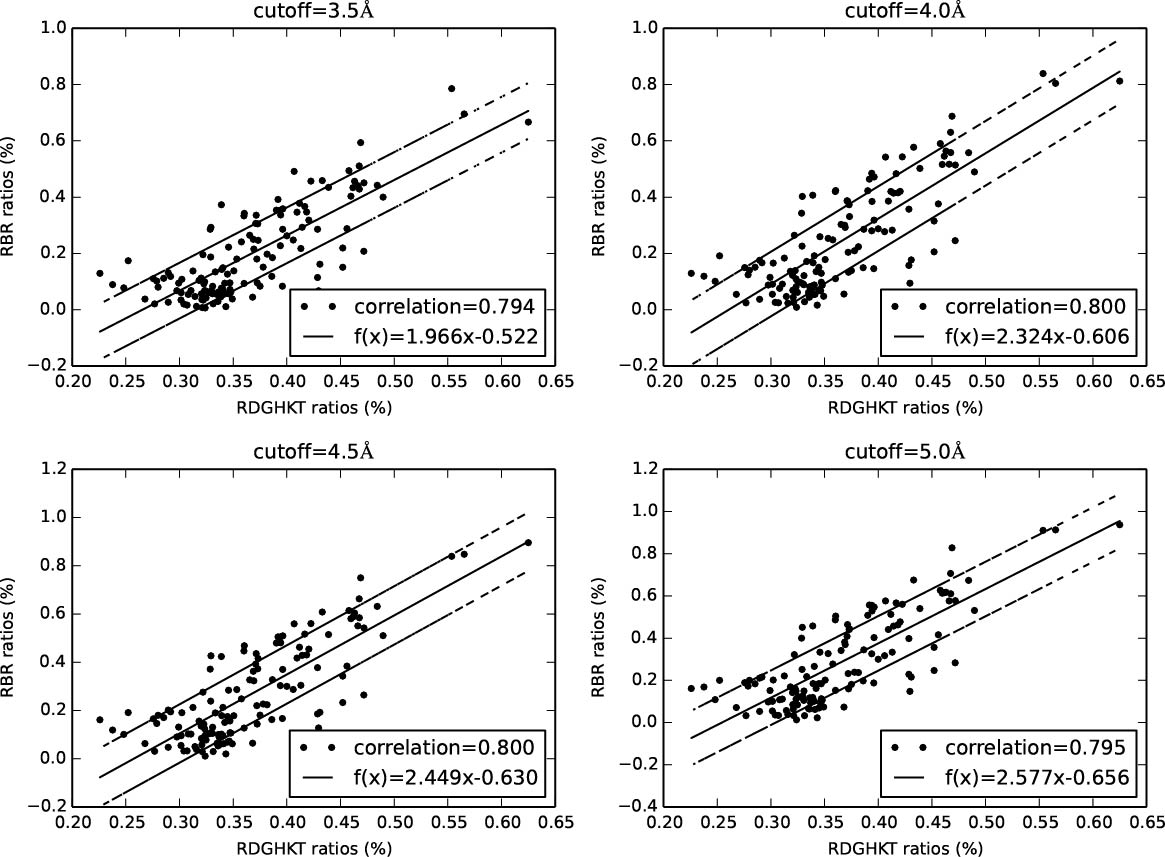


**Figure S4.** Correlation between the ratio of RDGHKT residues and the ratio of RNA binding residues in a protein. The linear function is shown in black and two dashed lines show the standard deviation region. Different distance cutoffs to define RNA binding sites were plotted. In the case of tRNA Pseudouridine Synthase B (PDB 1k8w, chain A), 97 out of 303 residues belong to the six types, giving a ratio of 0.32. The two standard deviation lines are f(x)=1.97x-0.52-0.1 and f(x)=1.97x-0.52+0.1. The maximum and minimum binding site numbers are calculated as (1.97×0.32-0.52-0.1)×303=3 and (1.97×0.32-0.52+0.1)×303=62, while the mean number is (1.97×0.32-0.52)×303=33. The real binding site number is 34, which is close to the mean number.


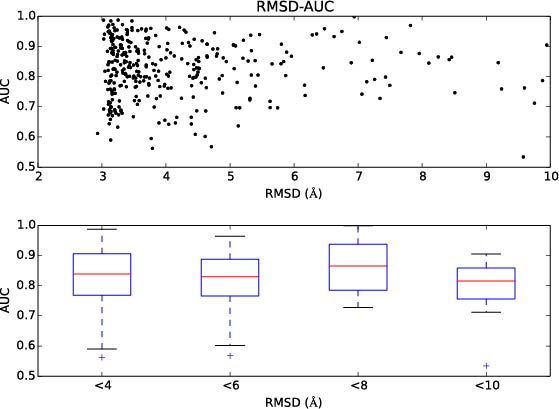


**Figure S5**. Dot plot and boxplot of the relationships between homology model quality (RMSD) and binding site prediction accuracy (AUC) on all 11 datasets of homologous structures. based on unbound states are far away from the crystallized complexed structure and we therefore exclude models worse than 10Å RMSD. The plots demonstrate that models more similar to the bound state structures (low RMSDs) offer a better performance, but the average AUC is always above 0.8.


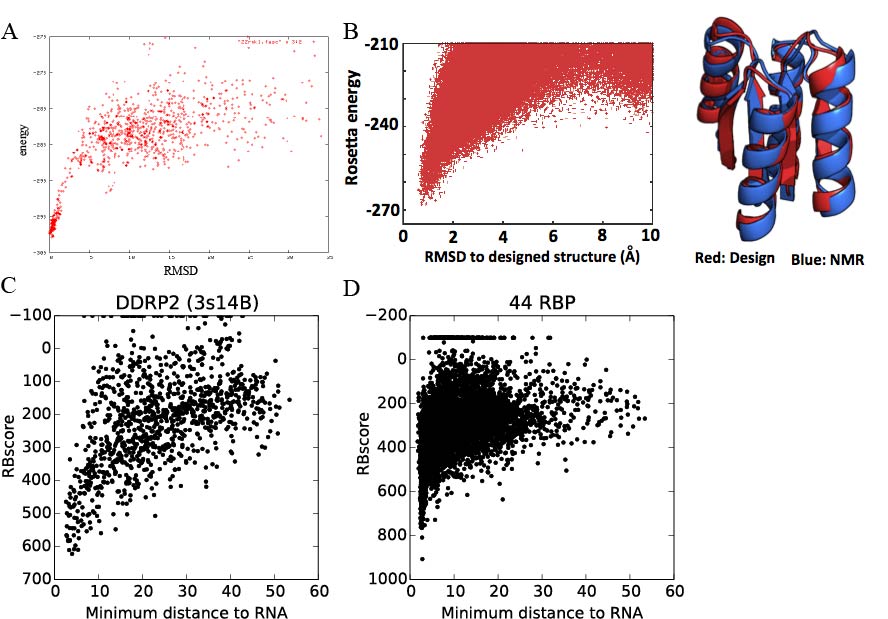


**Figure S6**. Energy funnel comparison between protein docking, protein folding and RBscore. A) Energy funnel from RosettaDock in protein-protein docking ([Lyskov & Gray, 2008](#_ENREF_13)). B) Energy funnel from RosettaDesign in protein folding ([Koga et al, 2012](#_ENREF_9)). C) Energy funnel of RBscore on DDRP2. D) Energy funnel of RBscore on a set of proteins.


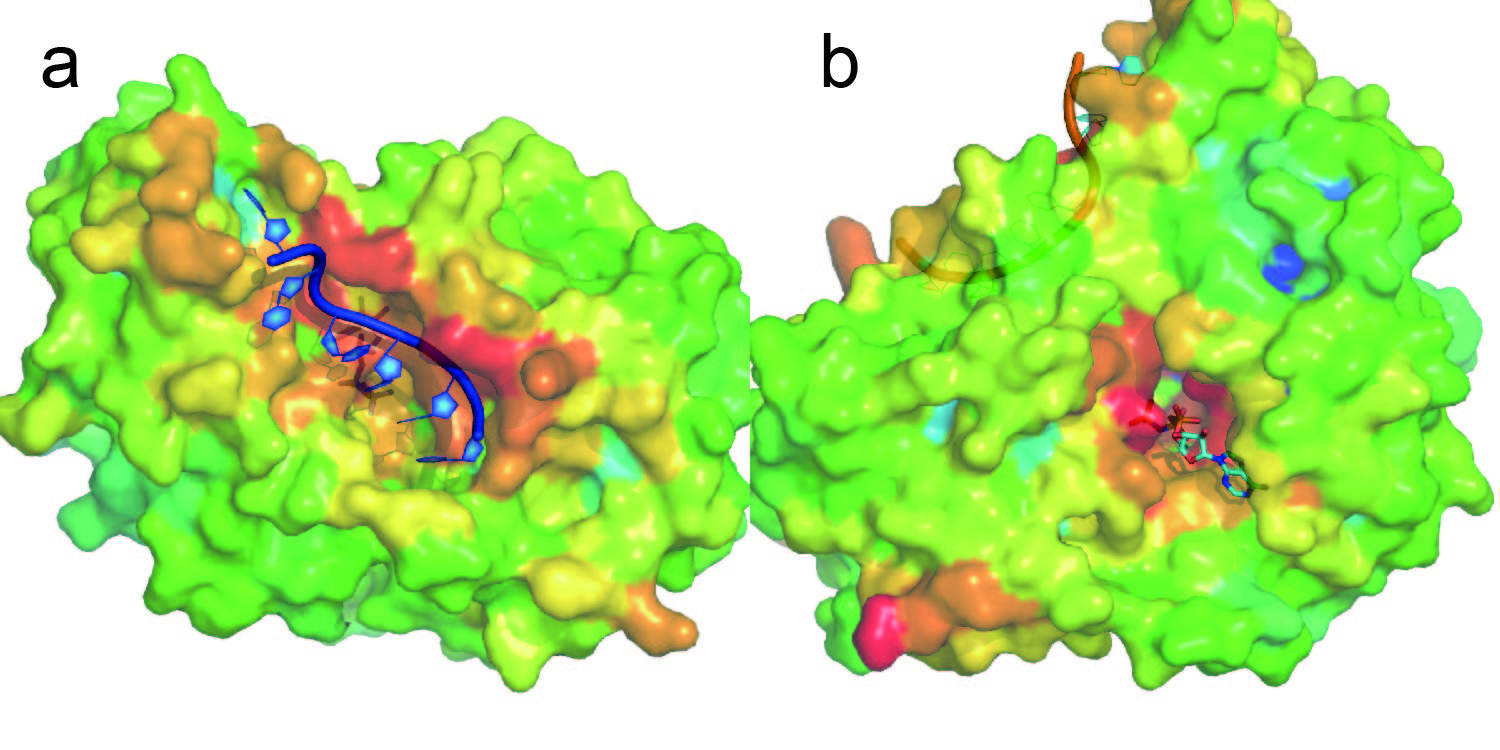


**Figure S7.** RBscore mapping on ATP-dependent RNA helicase DDX19 (PDB id 3G0H). a) The RNA binding region, b) the ATP-analogue (ANP) binding region.


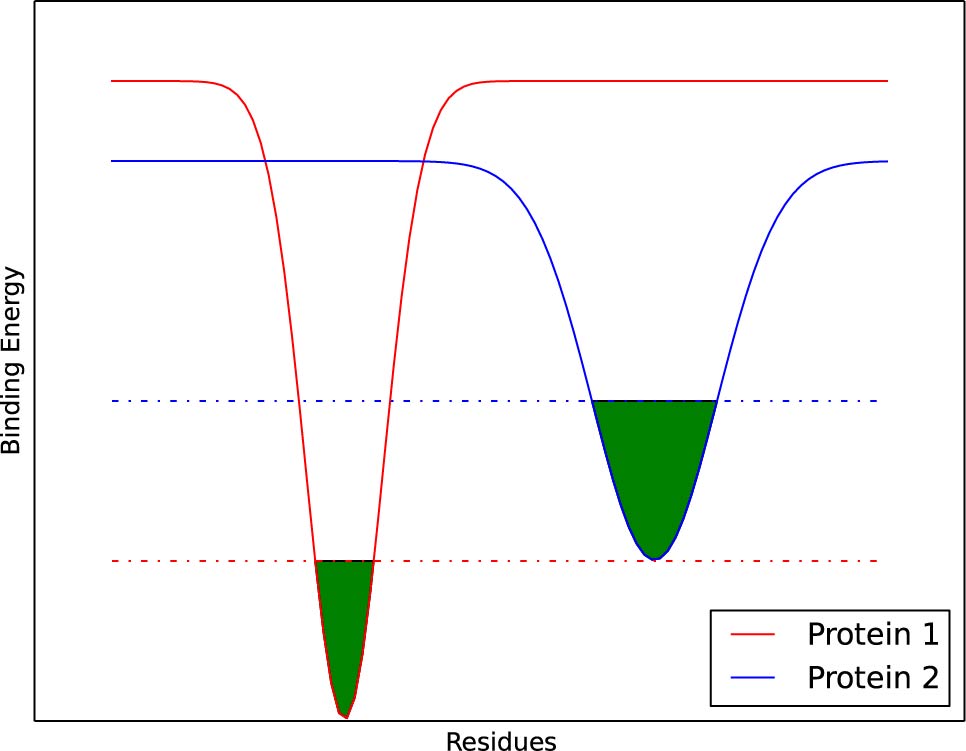


**Figure S8.** Illustration of the NA binding energy distributions on the accuracy as deduced by AUC. The red and blue lines show the distribution of NA binding affinities of residues in two different proteins. Dashed lines show two cutoffs for the binding sites, while the green regions are the binding sites. Since the two proteins have different affinities to NA, the energy cutoffs are different and should not be compared together. Otherwise, protein 1 would include false positive residues, while protein 2 includes false negative residues.


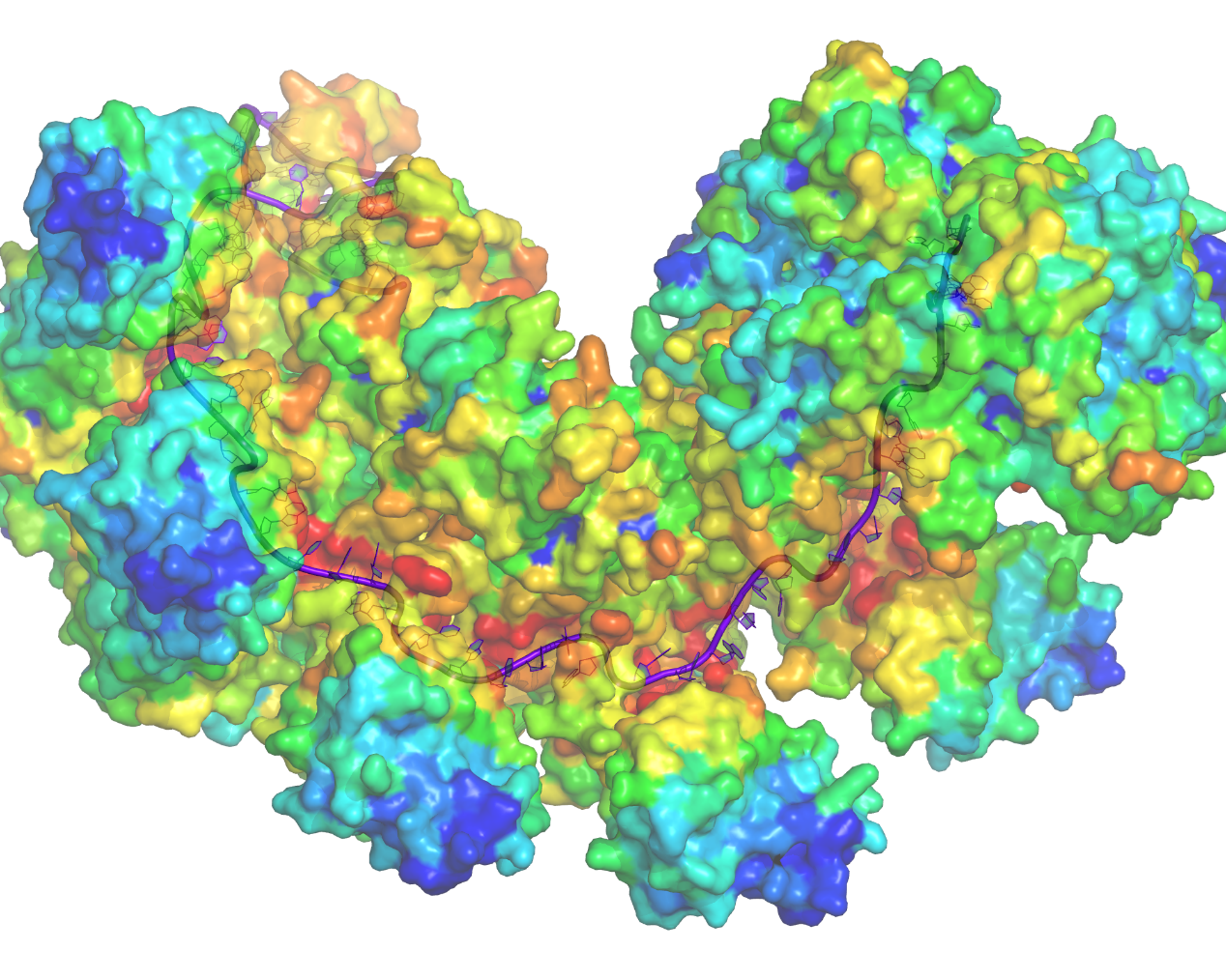


**Figure S9.** RBscore of RNA-guided Cascade complex([Zhao et al, 2014](#_ENREF_26)) mapped on structure with rainbow color. As each protein chain in the complex was predicted separately, the final map of RBscore still corresponds to exact binding region of RNA.


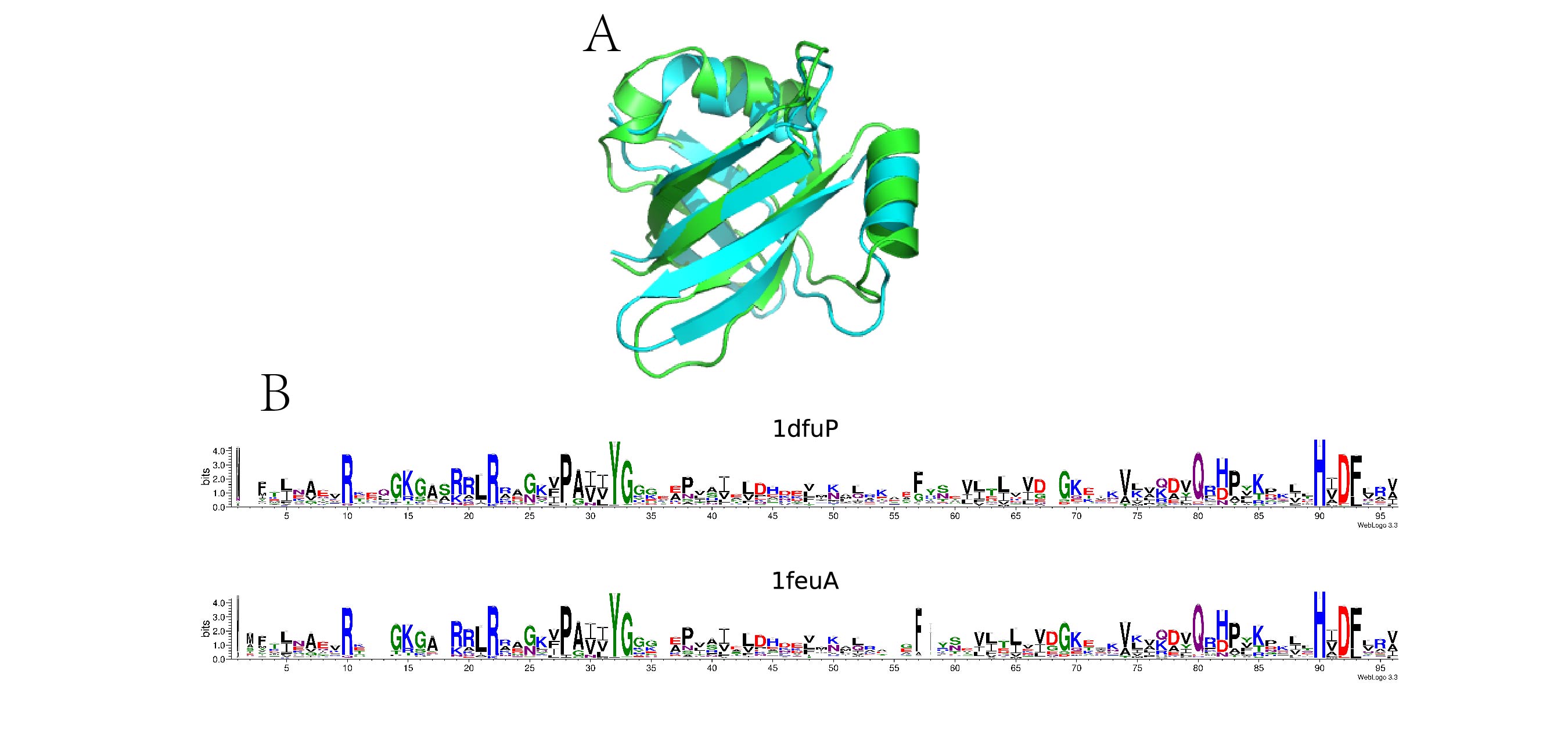


**Figure S10**. A) Superimposition between 1dfu chain P and 1feu chain A. B) sequence profiles of 1dfu chain P and 1feu chain A. Although sequence identity between the two proteins is low as 16.9%, they fold into similar structure and have similar sequence profiles.

**Reference**

Ahmad S, Gromiha MM, Sarai A (2004) Analysis and prediction of DNA-binding proteins and their binding residues based on composition, sequence and structural information. *Bioinformatics* **20:** 477-486

Allers J, Shamoo Y (2001) Structure-based analysis of Protein-RNA interactions using the program ENTANGLE. *J Mol Biol* **311:** 75-86

Berman HM, Westbrook J, Feng Z, Gilliland G, Bhat TN, Weissig H, Shindyalov IN, Bourne PE (2000) The Protein Data Bank. *Nucleic Acids Res* **28:** 235-242

Braga-Neto UM, Zollanvari A, Dougherty ER (2014) Cross-validation under separate sampling: strong bias and how to correct it. *Bioinformatics*

Cheng CW, Su ECY, Hwang JK, Sung TY, Hsu WL (2008) Predicting RNA-binding sites of proteins using support vector machines and evolutionary information. *Bmc Bioinformatics* **9**

Collins R, Karlberg T, Lehtio L, Schutz P, van den Berg S, Dahlgren LG, Hammarstrom M, Weigelt J, Schuler H (2009) The DEXD/H-box RNA Helicase DDX19 Is Regulated by an alpha-Helical Switch. *J Biol Chem* **284:** 10296-10300

Dunker AK, Lawson JD, Brown CJ, Williams RM, Romero P, Oh JS, Oldfield CJ, Campen AM, Ratliff CR, Hipps KW, Ausio J, Nissen MS, Reeves R, Kang CH, Kissinger CR, Bailey RW, Griswold MD, Chiu M, Garner EC, Obradovic Z (2001) Intrinsically disordered protein. *J Mol Graph Model* **19:** 26-59

Kim OTP, Yura K, Go N (2006) Amino acid residue doublet propensity in the protein-RNA interface and its application to RNA interface prediction. *Nucleic Acids Research* **34:** 6450-6460

Koga N, Tatsumi-Koga R, Liu G, Xiao R, Acton TB, Montelione GT, Baker D (2012) Principles for designing ideal protein structures. *Nature* **491:** 222-227

Kumar M, Gromiha AM, Raghava GPS (2008) Prediction of RNA binding sites in a protein using SVM and PSSM profile. *Proteins* **71:** 189-194

Li T, Li QZ (2012) Annotating the protein-RNA interaction sites in proteins using evolutionary information and protein backbone structure. *J Theor Biol* **312:** 55-64

Liu ZP, Wu LY, Wang Y, Zhang XS, Chen LN (2010) Prediction of protein-RNA binding sites by a random forest method with combined features. *Bioinformatics* **26:** 1616-1622

Lyskov S, Gray JJ (2008) The RosettaDock server for local protein-protein docking. *Nucleic Acids Res* **36:** W233-238

Ma X, Guo J, Wu JS, Liu HD, Yu JF, Xie JM, Sun XA (2011) Prediction of RNA-binding residues in proteins from primary sequence using an enriched random forest model with a novel hybrid feature. *Proteins* **79:** 1230-1239

Moult J (2008) Comparative modeling in structural genomics. *Structure* **16:** 14-16

Terribilini M, Lee JH, Yan CH, Jernigan RL, Honavar V, Dobbs D (2006) Prediction of RNA binding sites in proteins from amino acid sequence. *Rna* **12:** 1450-1462

Terribilini M, Sander JD, Lee JH, Zaback P, Jernigan RL, Honavar V, Dobbs D (2007) RNABindR: a server for analyzing and predicting RNA-binding sites in proteins. *Nucleic Acids Research* **35:** W578-W584

Tjong H, Zhou HX (2007) DISPLAR: an accurate method for predicting DNA-binding sites on protein surfaces. *Nucleic Acids Research* **35:** 1465-1477

Treger M, Westhof E (2001) Statistical analysis of atomic contacts at RNA-protein interfaces. *J Mol Recognit* **14:** 199-214

Walia RR, Xue LC, Wilkins K, El-Manzalawy Y, Dobbs D, Honavar V (2014) RNABindRPlus: A Predictor that Combines Machine Learning and Sequence Homology-Based Methods to Improve the Reliability of Predicted RNA-Binding Residues in Proteins. *PLoS ONE* **9:** e97725

Wang CC, Fang YP, Xiao JM, Li ML (2011) Identification of RNA-binding sites in proteins by integrating various sequence information. *Amino Acids* **40:** 239-248

Wang LJ, Brown SJ (2006) BindN: a web-based tool for efficient prediction of DNA and RNA binding sites in amino acid sequences. *Nucleic Acids Research* **34:** W243-W248

Wang LJ, Huang CY, Yang MQ, Yang JY (2010) BindN plus for accurate prediction of DNA and RNA-binding residues from protein sequence features. *Bmc Syst Biol* **4 Suppl 1:** S3

Wang Y, Xue Z, Shen G, Xu J (2008) PRINTR: Prediction of RNA binding sites in proteins using SVM and profiles. *Amino Acids* **35:** 295-302

Yang XF, Li HT, Huang YY, Liu SY (2013) The dataset for protein-RNA binding affinity. *Protein Sci* **22:** 1808-1811

Zhao H, Sheng G, Wang J, Wang M, Bunkoczi G, Gong W, Wei Z, Wang Y (2014) Crystal structure of the RNA-guided immune surveillance Cascade complex in Escherichia coli. *Nature* **advance online publication**
